# Self‐Assembly of Heterochiral and Homochiral Peptides Conjugated to PNA Dimers and Their Integration in Peptide‐Based Hydrogels

**DOI:** 10.1002/chem.202502255

**Published:** 2025-08-13

**Authors:** Ilaria Miglioli, Alessandro Ajò, Stefano Di Ciolo, Marco Carofiglio, Daria di Prisco, Dritan Siliqi, Luisa De Cola, Alessandra Romanelli

**Affiliations:** ^1^ Department of Pharmaceutical Sciences University of Milan Milano 20133 Italy; ^2^ Department of Molecular Biochemistry and Pharmacology Istituto di Ricerche Farmacologiche Mario Negri IRCCS Milano 20156 Italy; ^3^ Department of Applied Science and Technology Politecnico di Torino Torino 10129 Italy; ^4^ Institute of Crystallography CNR Bari 70126 Italy

**Keywords:** assembly, chirality, hydrogel, peptide, peptide nucleic acids

## Abstract

Hybrid biomolecules composed of short peptides and nucleic acid analogues can self‐assemble and are good candidates to produce new materials thanks to their biocompatibility and versatility. The assembly occurs by exploiting hydrogen bonds and stacking interactions between nucleobases and amino acids. The organization of the molecules at the supramolecular level is dictated by the structure of the assembling units. In this work we explored the effect of chirality on the self‐assembly of PNA‐peptide conjugates. We focused on homochiral and heterochiral peptides conjugated to PNA dimers and we characterized by spectroscopic techniques the assembled objects. Furthermore, we investigated the ability of the conjugates to form hydrogels in combination with the peptides. The architecture of the assembled molecules in the hydrogels was investigated, as well as the ability of hydrogels to support cell growth. Interestingly, hydrogels formed by homochiral and heterochiral mixtures show pores of different size. In the hydrogels cells grow along a specific direction, dictated by the fiber axis formed by the assembled compounds and their viability seems related to the gel pore size.

## Graphical abstract

Homochiral PNA‐peptide conjugates mixed to homochiral peptides yield hydrogels supporting cell growth.

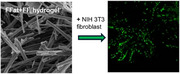

## 1 Introduction

The self‐assembly of biomolecules such as peptides or nucleic acids represents a valid strategy to produce biomaterials, thanks to their biocompatibility and low toxicity.^[^ ^1^ ^]^ Peptides, proteins, and nucleic acids assemble in different 3D structures, whose features are dictated by the sequence, the length of the molecules, or the protocol employed to promote their assembly. The combination of peptides and DNA in a single molecule was recently explored, with the aim to synergize the structural programmability of DNA and the chemical versatility of peptides.^[^ ^2^ ^]^

In principle it is possible to exploit interactions between peptides to force nucleic acids in a particular structure or even the other way around. As an example, coiled‐coil peptides conjugated to oligonucleotides have been employed to produce DNA origami.^[^ ^3^ ^]^ Alternatively, it is possible to use the interaction between peptides and nucleic acids in synergy, in such a way that both molecules contribute to the stabilization of a certain supramolecular structure. For example, conjugation of the dipeptide FF to a 12‐mer polypyrimidine oligonucleotide induces formation of hollow spherical structures, that could not be formed by mixing the two isolated components. Concentric bilayers are obtained by combining the (EF)n peptide conjugated to complementary DNA sequences.^[^ ^4^ ^]^ Nanospheres endowed with temperature‐dependent size controllability are produced using DNA conjugated to elastin‐like polypeptides.^[^ ^5^ ^]^ Many more examples can be found in the literature in which biomolecules can be programmed to achieve specific self‐assembly behavior.^[^ ^2^ ^]^ However, despite beautiful examples of supramolecular structures obtained by combining nucleic acids and peptides, some challenges still remain. In hybrid molecules, the interactions between nucleic acids and amino acids triggering self‐assembly are not easily encoded, therefore it is not straightforward to anticipate the 3D structure that will be obtained upon assembly based on the sequence.

Recently, we and others investigated the self‐assembly of hybrids composed of short peptides and PNAs.^[^ ^6^ ^]^ In many cases it was demonstrated that the peptide triggered aggregation and the morphology of the aggregates changed with the composition of the nucleobases. The conjugation of FF to PNA monomers or homodimers produced compounds capable of self‐assembling into fibers; when the PNA dimer “gc” was conjugated to the peptide FF, the morphology of the aggregates appeared spherical but the dimensions of the spheres were not homogeneous.^[^ ^7^ ^]^ When a longer peptide, FFFF, was conjugated to the PNA gc, spherical aggregates also formed. However, when FFFF was linked to the PNA dimer “at” intertwined fibers were observed.^[^ ^8^ ^]^ In the examples mentioned so far, the peptide drives the aggregation. Reasonably, in FFFF at the hydrogen bonds between the nucleobases zip the peptides, oriented in an antiparallel fashion.

The formation of fibers rather than spheres likely depends on a complicated interplay between steric and electronic factors, which involves aromatic moieties such as nucleobases and phenyl on the amino acid side chains. Recent studies carried out on short peptides with aliphatic and aromatic amino acids demonstrate that the repulsion between aromatic side chains and the backbone determines the twisting direction of beta strands that assemble into multilayered beta sheets and ultimately dictates the chirality of the final supramolecular structure.^[^ ^9^ ^]^ Additionally, in tripeptides it has been shown that the chirality of the amino acids affects the supramolecular organization of the assembled peptides: homochiral and heterochiral short peptides assemble to produce structures with different features.^[^ ^10^ ^]^ It was shown that heterochiral peptides such as Phe‐^D^Leu‐Phe, unlike homochiral peptides, arrange in a kinked beta strand conformation, in which the aromatic rings of Phe lie on the same side and create a hydrophobic region together with the Leu side chain, capable of excluding water.^[^ ^10b^ ^]^ As a consequence, segregation of hydrophobic and hydrophilic regions occurs and results in self‐assembly in water. Studies have also been reported on heterochiral dipeptides. Diphenylalanine peptides self‐assemble in nanotubes; the chirality of the N‐terminal amino acids dictates the helical arrangement.^[^ ^10a^ ^]^ Homochiral FF peptides assemble to produce tubular double layers of peptides, in which interdigitation between aromatic rings secures hierarchical assembly into bundles; fF heterochiral peptides do not zip, due to a higher level of intramolecular aromatic interactions (face to face interactions of Phe side chains) versus intermolecular. Heterochiral compounds produce fibrillar structures.

PNA monomers are not chiral; it is known that the conjugation of an amino acid to a PNA oligomer induces chirality in the PNA oligomer, which arranges in a right‐ or left‐handed helix with a D or a L amino acid at its termini.^[^ ^11^ ^]^ The effect of chirality on the self‐assembling ability of peptide‐PNA conjugates has not been explored so far. A change in the chirality of one amino acid could determine a change in the orientation of the aromatic moieties and therefore potentially could result in different 3D structures.

In order to explore the effect of amino acid chirality on the formation of supramolecular structures of PNA‐peptide conjugates, here we report a family of molecules in which the PNA dimer “at” is conjugated at the C‐terminus of the dipeptide FF (with the amino acids in the same L‐configuration) or to fF or to Ff, that is, peptides with the amino acids of opposite chirality. We studied the self‐assembly of homo and heterochiral peptide‐PNA conjugates by spectroscopic techniques.

Finally, we investigated the ability of the conjugates, either alone or in mixture with the peptides FF, fF or Ff, to form hydrogels. To explore the application of hydrogels as biomaterials, we also tested the hydrogels’ ability to support cell growth.

## 2 Results and Discussion

### 2.1 Design and Synthesis

**Figure 1:**
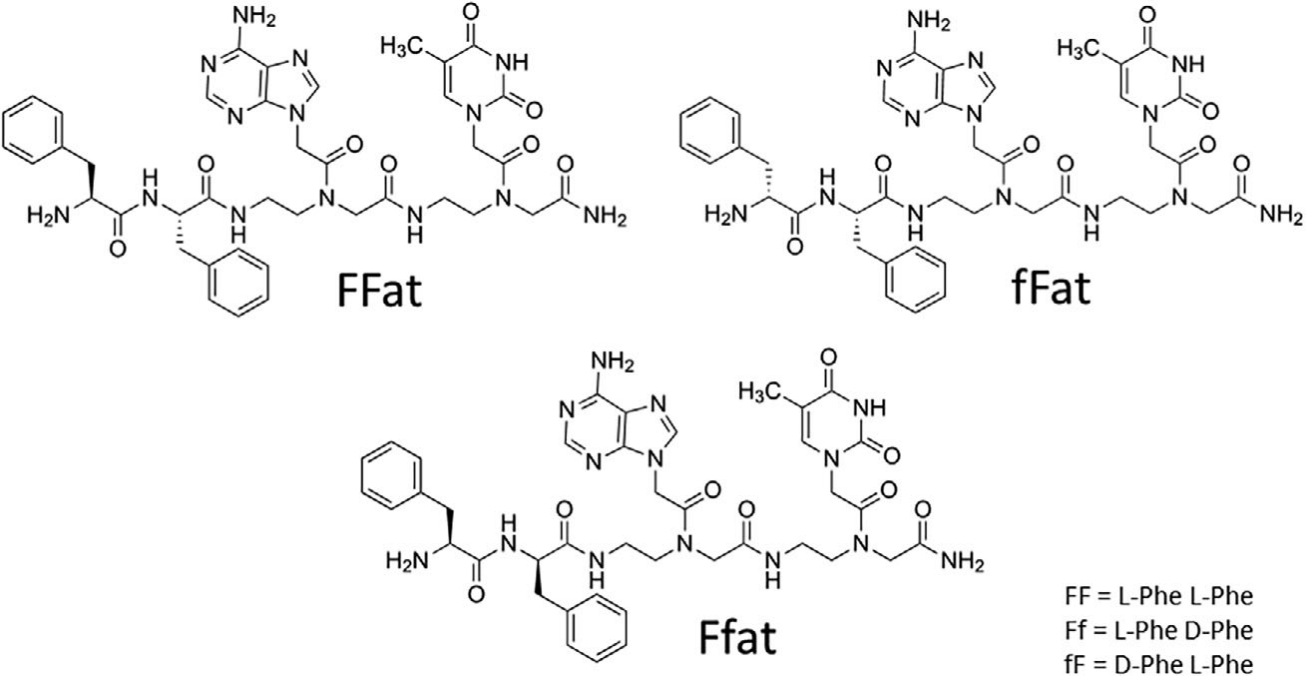
Chemical structure of the synthesized molecules.

The chemical structures of the molecules here synthesized and investigated are reported in Figure 1.

**Figure 2:**
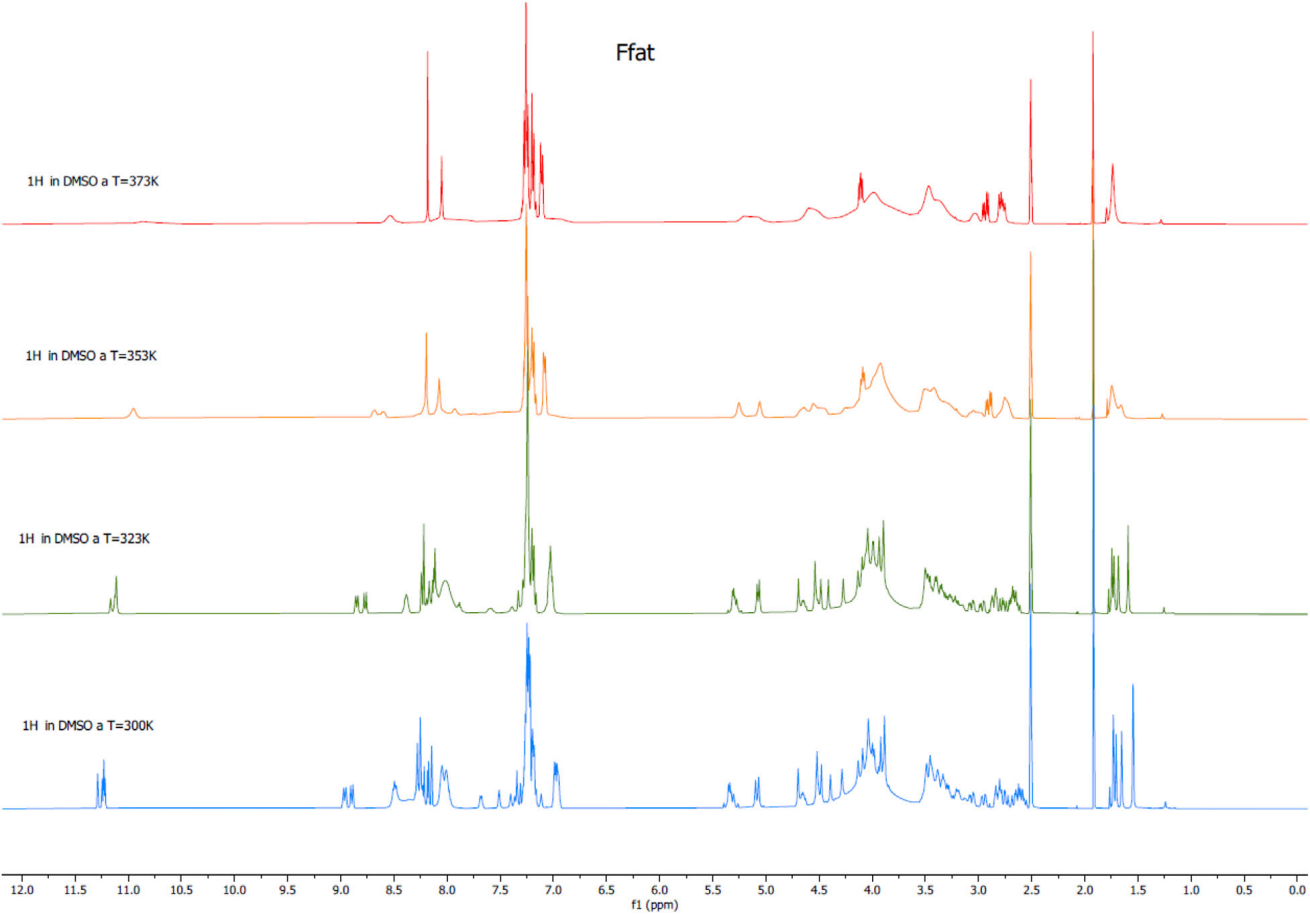
Variable temperature NMR spectra of the Ffat conjugate.

In all molecules peptides are at the N‐terminus and the C‐terminus is amidated. The relative position of the peptide and the PNA was chosen based on previous work on conjugates of diphenylalanine to the PNA dimer gc, suggesting a higher propensity to aggregation when the peptide is at the N‐terminus versus C‐terminus position.^[^ ^7^ ^]^ Moreover, no differences in the aggregation propensity and morphology of the assembled compounds were observed comparing PNA‐peptide conjugates with an amide and a carboxyl C‐terminal end. The PNA sequence was chosen self‐complementary to promote assembly through hydrogen bonds between complementary nucleobases. Unlike “gc” the “at” dimer has already been shown to promote formation of ordered aggregates when conjugated to a tetraphenylalanine peptide.^[^ ^8^ ^]^ All compounds were obtained by solid‐phase synthesis, using standard protocols for Fmoc chemistry. Compounds were purified by RP‐HPLC and characterized by ESI‐MS, mono, and bi‐dimensional NMR (Figures S1–S3, S17–S34, and Tables S1–S3). The spectra of the PNA‐peptide conjugates appear quite complicated due to the free rotation of the methylene carbonyl linker between the nucleobase and the backbone, resulting in different rotamers (cis and trans). The existence of rotamers interconverting at room temperature is described in the literature for standard achiral PNA monomers as well as for chiral PNA monomers.^[^ ^12^ ^]^ For a sequence of N residues, there are 2 ^N^ possible rotamers; in our case 4 possible rotamers can exist. To prove the existence of rotamers, ^1^H NMR experiments at variable temperature were carried out on one sample (Figure 2). A broadening of signals was observed at increasing temperatures, consistent with what reported in the literature for systems of similar complexity.^[^ ^12b^ ^]^

### 2.2 Fluorescence Studies

The critical aggregation concentration (CAC) of the conjugates was determined by fluorescence spectroscopy, monitoring the emission of the 1‐anilino‐8‐naphthalene sulfonate (ANS) at 490 nm.^[^ ^13^ ^]^ The ANS fluorescence is sensitive to the polarity of the environment and for this reason it is used to monitor aggregation of peptides and PNA‐peptide conjugates.^[^ ^6^, ^14^ ^]^ Increasing concentrations of the PNA‐peptide conjugates were titrated into ANS solution; an abrupt increase in the fluorescence emission intensity is observed when the aggregation process occurs. The CAC values calculated are similar for the three compounds, being lower for the conjugates of PNA to heterochiral peptides (Table 1 and Figure S4). Lower CAC values are observed for compounds exhibiting a higher retention time. This observation is consistent with what is reported in the literature for self‐assembling peptides.^[^ ^10b^ ^]^ Interestingly in this case amino acid chirality determines a difference in the hydrophobicity of the peptides; in‐fact the homochiral FFat is eluted at a lower retention time than the heterochiral fFat and Ffat. The CAC value found for the homochiral is higher as compared to that reported for similar conjugates, in which the PNA “gc” replaces the “at”. The difference in the CAC could be attributed to the different hydrogen bonding ability of gc versus at; the higher number of hydrogen bonds of the couple gc could determine a more pronounced ability to self‐assemble.The difference in the CAC of homochiral and heterochiral compounds may be ascribed to a different strength in the interactions between assembling molecules, resulting in a different molecular packing of the compounds. We might speculate that in the heterochiral compounds the Phe side chains are on the same side and might be involved in intramolecular face to face interactions, similar to those reported for the heterochiral diphenylalanine peptides described by Marchesan.^[^ ^10a^ ^]^ Different monomers might sandwich one on top of the other due to interactions between the Phe aromatic rings, while the nucleobases are far apart. In the homochiral compounds, both nucleobases and phenyl rings interactions stabilize the structure, likely the phenyl rings are not one on top of the other but rather on a more extended structure, characterized by a lower number of aromatic interactions or a combination of hydrogen bonds and aromatic interactions. The difference in the number and type of interactions might explain the difference in the CAC (Table 1).

**Table 1:** Retention time of conjugates analysed by RP‐HPLC and CAC.

Compound name	Retention time (min) in RP‐HPLC	CAC (mM)
FFat	10.2	1.51
fFat	12.7	0.57
Ffat	12.8	0.52

The solubility of the conjugates in water and in phosphate buffer is comparable, around 40 mM. In buffer at 40 mM the compounds precipitate after few hours.

**Figure 3:**
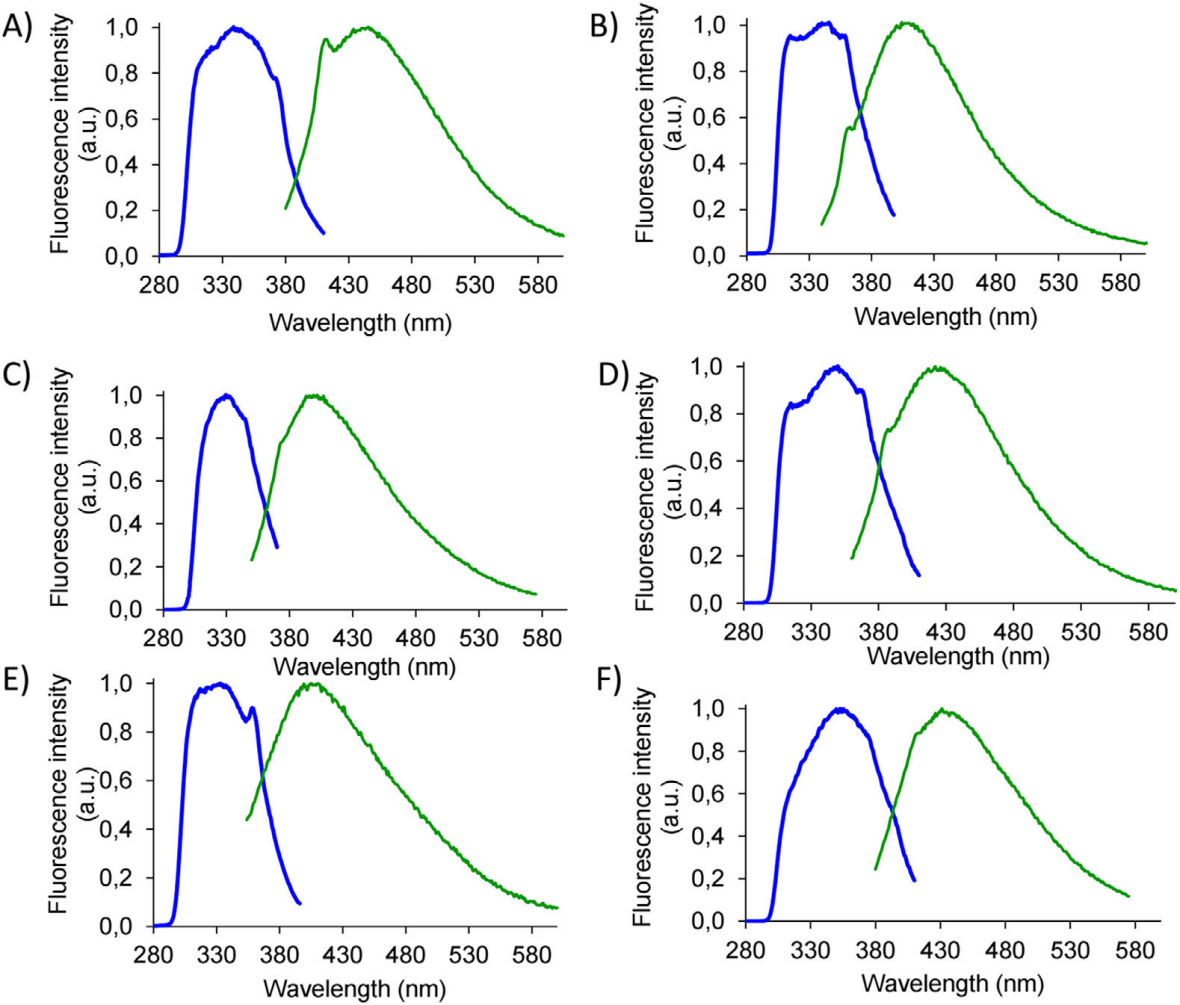
Fluorescence spectra of self‐assembled compounds in phosphate buffer 0.1 M, pH 7.4 at 10X CAC. Blue curves are the excitation spectra and green curves are the emission spectra. Excitation and emission wavelengths (*λ*_ex_ and *λ*_em_) are specified for each compound. A): FFat *λ*_ex_ = 320 nm and *λ*_em_ = 410 nm; B): FFat *λ*_ex_ = 340 nm and *λ*_em_ = 420 nm; C) Ffat *λ*_ex_ = 330 nm and *λ*_em_ = 390 nm; D): Ffat *λ*_ex_ = 360 nm and *λ*_em_ = 430 nm; E): fFat *λ*_ex_ = 310 nm and *λ*_em_ = 410 nm; F): fFat *λ*_ex_ = 360 nm and *λ*_em_ = 430 nm.

The fluorescence emission of the aggregated compounds was investigated at pH 7.4 in phosphate buffer (Figure 3). Aggregation spontaneously occurs when samples are dissolved in buffer. All compounds do not emit upon excitation at 257 nm, but in all cases, we observe emission between 390 and 410 nm upon excitation at 320–330 nm and emission around 420–430 upon excitation at 340–360 nm. The emission is likely due to excimers, that is, species generated in the excited state; in fact, bands in the excitation spectra are not visible in the absorption spectra. Similar features were observed for other PNA‐peptide conjugates and were attributed, respectively, to nucleobase aggregation and to hydrogen bond formation between the backbones.^[^ ^6a^ ^]^ As fluorescence is due to aggregation, plotting the fluorescence intensity at 390 or 410 nm versus concentration allows us to calculate again the CAC. Values calculated in this way are comparable to those obtained by ANS titration. (Figure S5)

The emission of fluorescence was monitored for all compounds in the aggregated form at different wavelengths. For FFat and Ffat, we observed an increase in the emission wavelength upon increasing the excitation wavelength (Figure S6). This phenomenon, named REES, already reported for aggregated PNA dimers is observed when a polar sample is in a polar solvent.^[^ ^15^ ^]^ The molecules are not oriented in a random fashion, different states characterized by different energy gaps between the ground and excited states with different relaxation times are present and this results in red‐shifted absorption and emission. The fact that only two aggregated compounds exhibit REES suggests a different organization of the building blocks in the supramolecular aggregates, with conjugates having L‐Phe at the N‐terminus showing a similar behavior.

### 2.3 Secondary Structure Studies

**Figure 4:**
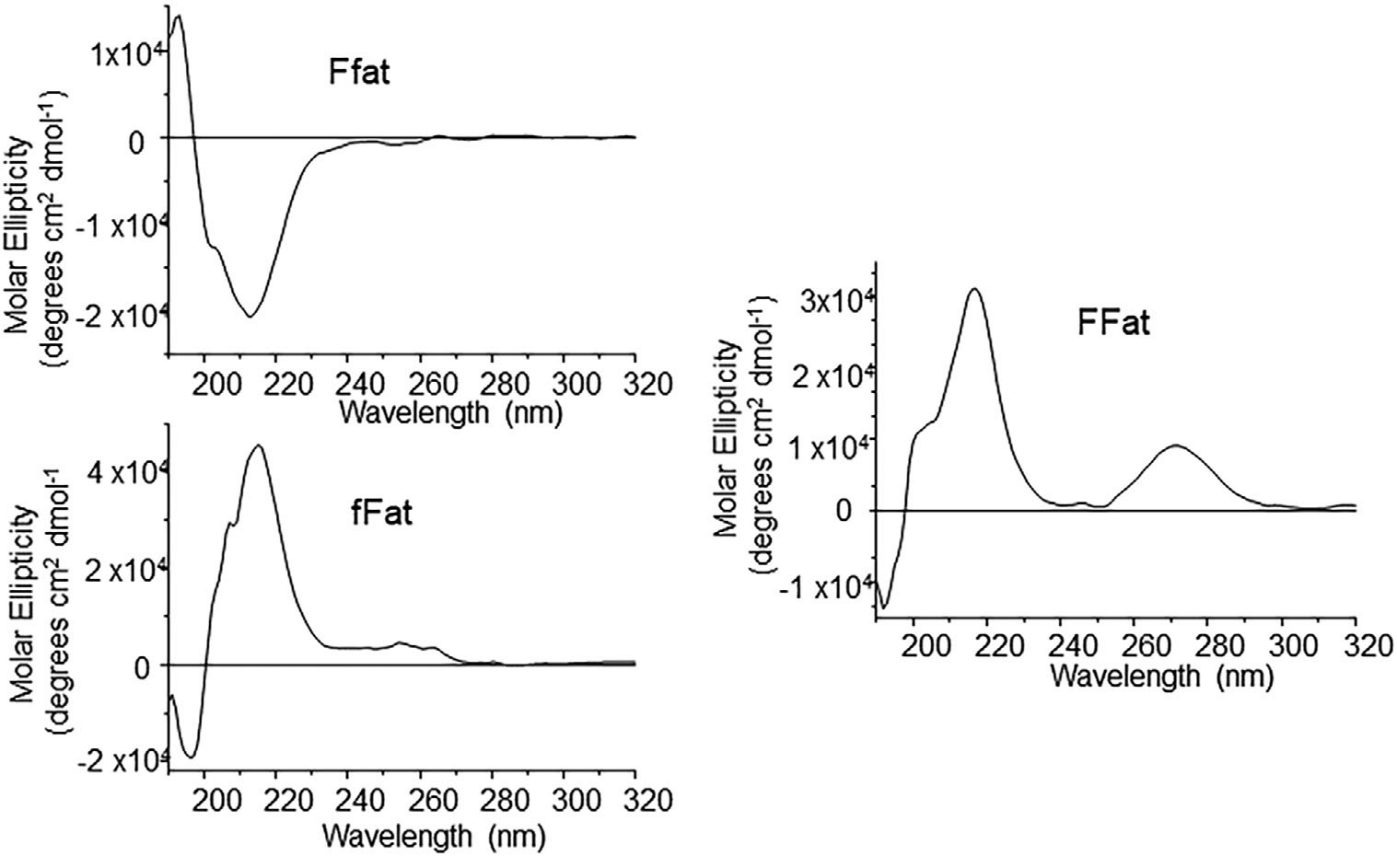
CD spectra of indicated peptide‐PNA conjugates at 5x CAC in phosphate buffer 0.1 M, pH 7.4.

To detect the secondary structure of the aggregated compounds, we performed Circular Dichroism experiments on our compounds in phosphate buffer, pH 7.4 at concentration 5x CAC (Figure 4). The spectrum of FFat shows an intense positive band centered around 218 nm, with a shoulder around 200 nm, and another absorption around 270 nm. The first band and the shoulder can be attributed to n‐π* and π‐π* transitions of the peptide backbone, the second is due to nucleobases stacking. A similar CD spectrum is reported for self‐assembled WWgc, in which π‐π aromatic interactions between the amino acids side chains in two facing monomers oriented in an antiparallel manner and Watson‐Crick hydrogen bonds between complementary nucleobases result in a right‐handed helical bilayer. When we observe the spectra of PNA conjugated to the heterochiral peptides, a pronounced band around 218 nm appears, while the signal around 270 nm is very low or absent, suggesting that stacking between nucleobases is weak. The band at 218 nm is opposite in sign for the heterochiral compounds. The chirality of the amino acid linked to the “a” PNA monomer determines the chirality of the molecule. CD data suggest a different organization of the supramolecular aggregates of homochiral and heterochiral PNA‐peptides. We speculate the formation of a helical bilayer similar to that observed in WWgc for the homochiral compound, stabilized by interactions between nucleobases and aromatic side chains of two different monomers. In the heterochiral conjugates, as observed in the heterochiral peptide reported in the literature, the aromatic side chains of the peptide lie on the same side; it may be that the aggregates are stabilized by interactions between aromatic side chains of amino acids of two different monomers in a head to head arrangement (N terminus ‐N‐terminus), while the PNA lies at C‐terminal end far apart from one another.

With the aim to characterize the morphology of our aggregates, we performed AFM experiments on the aggregates. In all cases we detected formation of irregular objects (data not shown).

### 2.4 Hydrogel Preparation

The ability of PNA‐peptide conjugates to form hydrogels was investigated. In the literature hydrogel formed by PNA‐peptide conjugates were observed in two cases: amphipathic peptides conjugated to PNA monomers or hydrophobic peptides.^[^ ^6^, ^14^, ^16^ ^]^ Hydrophobic peptides conjugated to PNA monomers are reported to form hydrogels, that can be applied as CEST‐MRI agents.^[^ ^14a^ ^]^

The peptides FF and fF are reported to form hydrogels at 20 mM concentration, in phosphate buffer at a neutral pH.^[^ ^9^ ^]^ Gelification experiments were initially attempted at pH 7.4 in buffer on all three compounds (FFat, fFat, and Ffat), at concentrations up to 40 mM; samples were heated to 90 °C and slowly cooled down at room temperature. No gel was formed; samples precipitated from the solution.

In a different set of experiments, we investigated the formation of hydrogel by mixing peptides and PNA‐peptide conjugates in different molar ratios. We mixed FF with FFat, fF with fFat and Ff with Ffat. We will refer to the FF + FFat mixture as FFmix, to the fF + fFat mixture as fFmix and to the Ff + Ffat mixture as Ffmix. We hypothesized that the PNA‐peptide conjugates could “intercalate” between the peptides forming nanotubes exploiting the diphenylalanine moiety and the nucleobases point outward the tube and interact with other nucleobases on other tubes, crosslinking the nanotubes.

We tested gelification of mixtures of peptides and peptides‐PNA conjugates in different w/w ratios at pH 7.4 in buffer. We found that the minimal concentration of peptide in the peptide/peptide‐PNA conjugate mixture able to produce hydrogel is 30 mM. Incorporation of the PNA‐peptide conjugate in the hydrogels was verified by RP‐HPLC analysis (Figure S7). By increasing the concentration of the peptide, higher amounts of the peptide‐PNA conjugates could be added and afforded a hydrogel. We characterized hydrogels containing 1.5% w/v of peptide and 0.2% w/v of peptide‐PNA conjugates. This is the mixture containing the highest amount of peptide‐PNA conjugate that was able to form a hydrogel.

To ensure proper mixing, the gel was obtained by heating the peptide solution at 90 °C, adding the peptide‐PNA conjugate solution, and then cooling down to room temperature. The hydrogel forms immediately. The hydrogel can be heated and cooled down multiple times, and it consistently forms.

### 2.5 Hydrogel Characterization

Rheological analyses were performed on freshly prepared hydrogels. Based on the literature data on similar materials, we conducted preliminary strain sweep tests to identify the linear viscoelastic region (LVR) for our material.^[^ ^10^, ^17^ ^]^ The selected strain range was between 1 and 100 Pa. Within this range, the material exhibited linear behavior up to approximately 10 Pa. Beyond this point, the material became less responsive, and the storage modulus (G′) declined. A pronounced drop in G′ was observed around 60 Pa, indicating a loss of linearity, and the breakdown of the hydrogel structure, as evidenced by G′ falling below G″. Such a decrease in G′ is typical in amplitude sweep tests when the applied strain exceeds the LVR.

**Figure 5:**
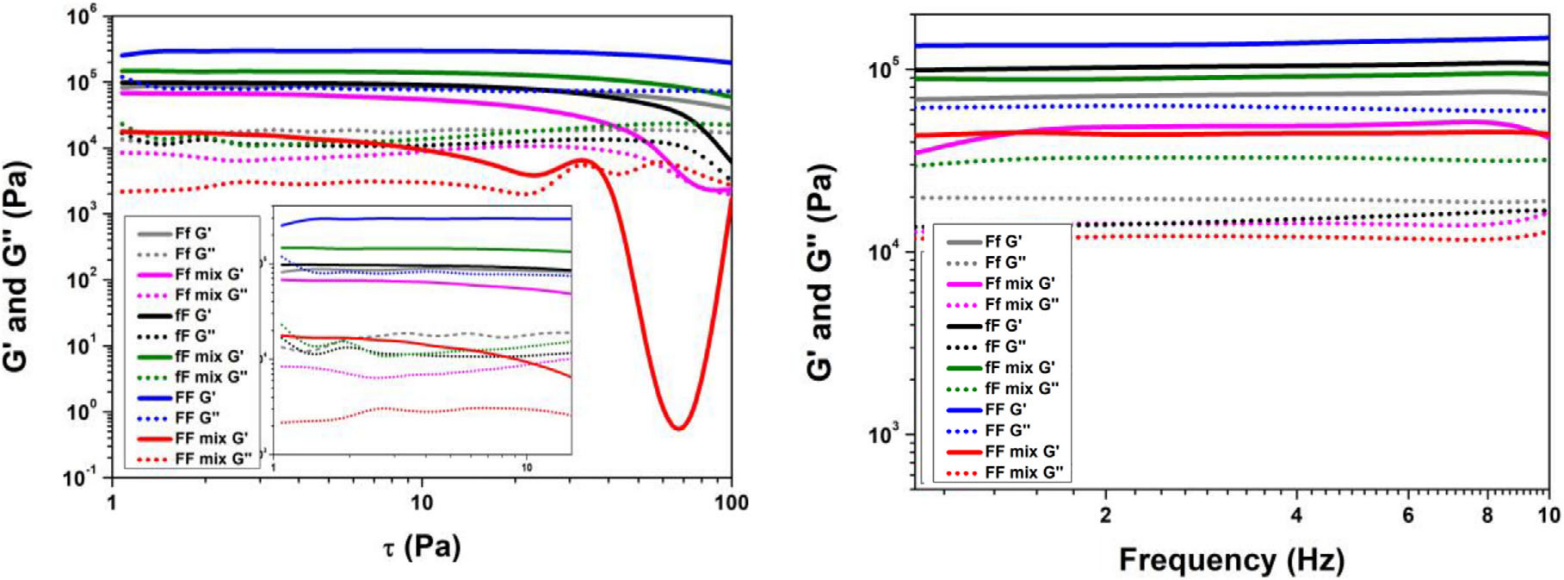
Amplitude sweep on the left and frequency sweep on the right of all hydrogels.

Amplitude sweep measurements were performed using a constant shear and sweep parameters between 0.46 Pa–100 Pa with a frequency of 0.1592 Hz. The resulting G’ values are presented in Table 2. Both the storage modulus (G’) and loss modulus (G‘’) were monitored across amplitude and frequency. For a solid gel, the storage modulus (G’) must exceed the loss modulus (G‘’) (G’ > G‘’), as shown in Figure 5. This behavior confirms that the material exhibits viscoelastic solid properties. For the peptides FF and fF, G’ values are slightly higher as compared of those reported in the literature, probably due to a difference in the concentration of the peptides.^[^ ^10a^ ^]^ The data indicate that incorporating heterochiral PNA‐peptide conjugates into heterochiral peptides does not change resistance under applied stress. Conversely, the mixture of homochiral PNA‐peptide conjugate with homochiral peptide exhibits a lower G’ value compared to the homochiral peptide alone. These findings suggest distinct organization in the components of the mixtures.

**Table 2:** Rheological characterization.

Sample	G’ mean (Pa) (x10^4^)	STDEV (x10^4^)
FF	25.0	7,5
FFmix	5.27	3,0
Ff	7.90	0.6
Ffmix	8.18	1,4
fF	8.60	3.0
fFmix	13.6	0.7

Abbreviation: STDEV: standard deviation.

Frequency sweep measurements were performed over a range of 0.1 Hz to 10 Hz with a strain of 0.1% to evaluate the material's behavior across different frequencies. To ensure valid and reliable results, the frequency sweep was conducted within the LVR, where the material remained responsive and exhibited linear behavior. The G’ values measured for hydrogels containing only the heterochiral peptide and those containing both the heterochiral peptide and heterochiral peptide/PNA conjugates are comparable, indicating that in all cases the material remains resistant to applied stress and strain.

In contrast, for homochiral compounds, the PNA‐peptide mixtures form hydrogels with a lower G’ value than the peptide alone, suggesting reduced resistance to stress. This reduction is attributed to the softer packing of the mixture, resulting in diminished mechanical properties, as both components share the same chiral center. Additionally, the measurement of G’ and G‘’ as functions of frequency demonstrates complete linearity within the same range of the LVR, without any crossover point (G’ = G‘’), confirming the material's structural integrity.

Amplitude sweep experiments (Figure 5) illustrate the LVR, confirming that the materials remain resistant up to 10 Pa under a constant strain applied at 0.1592 Hz. Similarly, the frequency sweep graphs on the right show no crossover between G’ and G‘’, further confirming the stability of the gel. A comparison of the amplitude and frequency sweep measurements reveals that the material's viscoelastic properties are both stable and reproducible across the tested range for both types of sweeps.

The fluorescence emission of hydrogels was investigated, placing a small piece of hydrogel into a quartz tube, and exciting at fixed a wavelength. Excitation wavelengths were chosen based on results observed in solution for the peptide‐PNA conjugates. For hydrogels formed with FFmix, fFmix, and Ffmix excitation wavelength was between 340 and 360 nm. Emission spectra of hydrogels with heterochiral compounds are all characterized by a main emission maximum around 570 nm and a lower intensity signal around 470 nm; for FFmix the emission maximum is observed around 450 nm. Lower intensity signals can be observed at higher wavelengths. (Figure S8)

X‐Ray Diffraction (XRD) analyses were performed on lyophilized hydrogels. The samples analyzed are the peptides (FF, fF, and Ff) and the mixtures (FFmix, Ffmix, and fFmix). The diffraction patterns (Figure S9) show the crystalline structure of the hydrogels; no significant differences between hydrogels formed by the peptides and the mixtures are observed. We can assume that the supramolecular architecture of gels is formed by assembled microcrystals.^[^ ^10a^ ^]^

**Figure 6:**
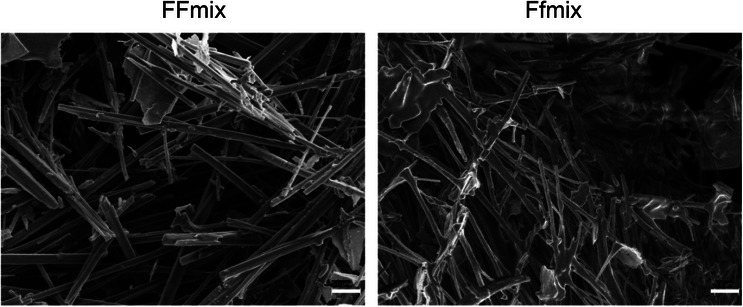
Scanning electron microscopy mages of lyophilized hydrogel composed of FFmix (on the left and Ffmix on the right. The scale bar reported in the picture is 5 µm.

Characterization was further carried out by SEM on lyophilized hydrogels. SEM images reveal the presence of tubular structures for peptide hydrogel. Hydrogels that contain the mixture of peptide and peptide‐PNA conjugate contain tubes of different lengths and widths, apparently shorter as compared to tubes observed in hydrogels formed by peptides. Tubules are densely interconnected. (Figure 6 and Figure S10).

### 2.6 SAXS Measurements

Finally, preliminary Small‐angle X‐ray scattering (SAXS) measurements were performed on the hydrogels and on lyophilized hydrogels (Figure S11). The observation of SAXS profiles on all samples confirms the presence of some elongated‐shaped objects (q^−^ slope) in the Guinier region, consistent with observations from the SEM images. To gain more details from SAXS, we analyzed the experimental data by using SasView v.6 software (https://www.sasview.org). For both types of samples (hydrogel and lyophilized hydrogel) two modelling‐fitting approaches were used: i) gel model ii) cylindrical and/or ellipsoid shape models.

For the gel model fitting the following parameters were fitted^[^ ^18^ ^]^:

**ξ** Corr‐length: Describes local fluctuations and equilibrium behavior.

**R_g_** Radius of Gyration: Accounts for static, larger‐scale polymer accumulations.

**Df** Fractal Dimension: Describes how the gel's structural density evolves across scales and connects these two characteristic lengths. In gels with significant hydrogen bonding, Df has been reported to be ∼2.6 to 2.8.

For shape model fitting using the observations from SEM images but considering the polydispersity, we used a mixed model of ellipsoids and cylinders refining the equatorial (R_eq_) and polar radius (R_p_) for the first and radius (R) and length (L) for the second.

**Figure 7:**
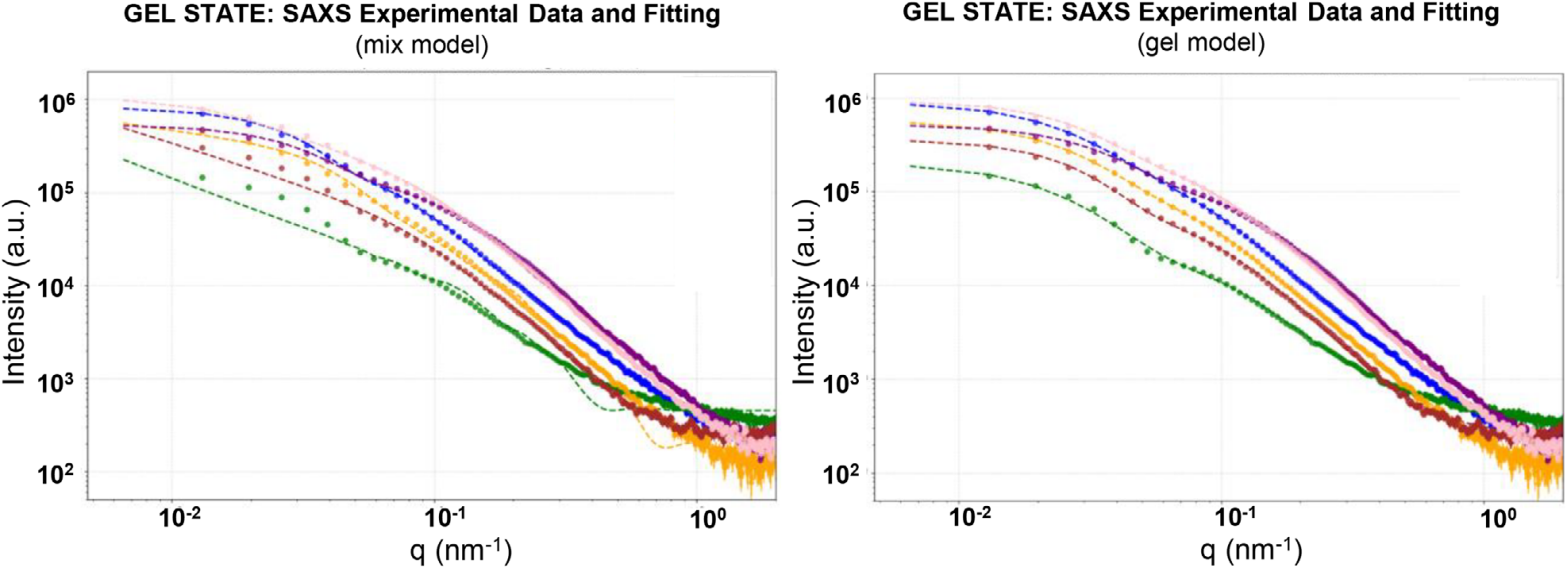
Hydrogel analysis with (left) a mixed model (combination of ellipsoid and cylinder components) and (right) a gel model. Both models are represented by dashed lines and are fitted to the experimental data, which are shown as filled circle symbols. Blue: FF, green: FFmix, yellow: Ff, purple: Ffmix, brown: fF, pink: fFmix.

All refined parameters for the gel state and lyophilized state are summarized in the Supplementary Tables S4–S7. The fitting for the analysis on the hydrogel is graphically represented in Figure 7. The fitting for the lyophilized samples is reported in Figure S12.

The results from the mixed model fitting on the hydrogels provide insightful details about the structural features of the samples. A high structural diversity across the samples is evident. Interestingly, an increase in the cylindrical shaped objects is observed in almost all cases in the mixtures of peptide and conjugates as compared to the peptides, in line with what is observed in the SEM images (Supplementary Figure S10). χ2 values suggest that the mixed model provides a robust framework for characterizing the structural features of the hydrogel‐forming assembled compounds, although extreme cases may require refinement or additional modeling considerations.

Additional insights were obtained using the gel model, which highlights distinct structural features across the samples, ranging from fine‐mesh gels to highly porous and coarse networks. Df values suggest a major contribution of weak interactions between the molecules that compose the hydrogel, likely interactions between aromatic building blocks. Hydrogels formed by heterochiral mixtures present medium pores, based on Correlation Length (ξ) values, while the hydrogel formed by the homochiral mixture presents large pores. Most samples have χ2 values below 2.0, indicating a reasonably good fit for the gel model. Slightly higher χ2 values for samples like Ff and Ffmix suggest potential structural complexities or irregularities not fully captured by the model.

### 2.7 Cell Growth

**Figure 8:**
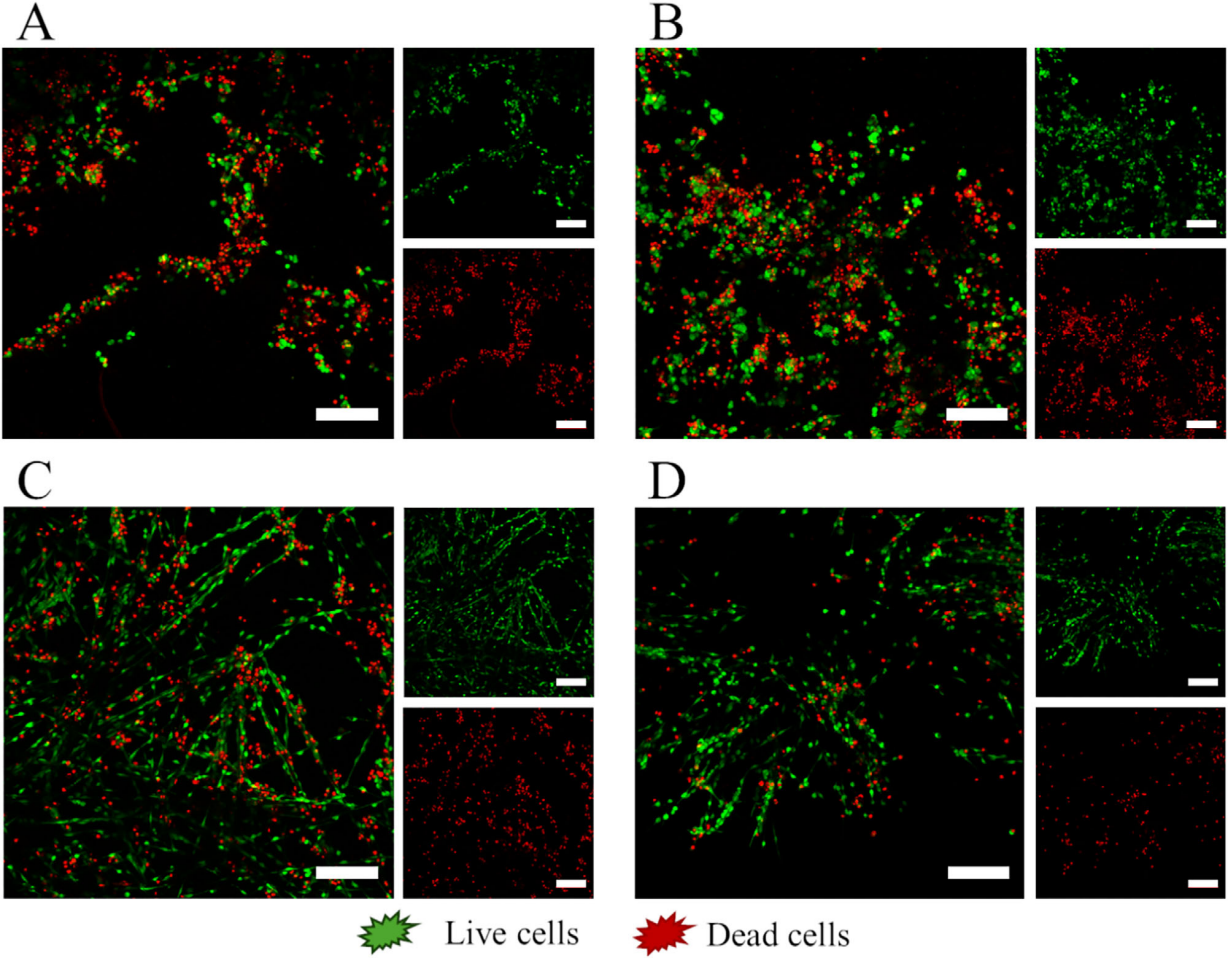
Live/Dead microscopy images of NIH/3T3 grown on A) fF, B) fFmix, C) FF, D) FFmix. Scalebar is 200 µm for all the images.

The ability of the hydrogels to support cellular growth was assessed through a Live/Dead assay. NIH/3T3 cells were seeded directly onto the hydrogels to determine whether they could attach to the hydrogel surface and proliferate (Supplementary Video). Figure 8 shows the results of the Live/Dead assay after 24 hours. The images represent a maximum fluorescence intensity projection across a depth of approximately 700 µm. This projection enables the visualization of 3D structures that would not be discernible from a single slice of the acquired image. A 3D reconstruction of the cells growing within the gels throughout the space is available in the Supporting Videos (Video 1, 2).

As can be noticed, cells grown on fF and fFmix exhibit a spherical morphology, typically associated with poor adhesion to the substrate. Despite this, some live cells are still able to grow on top of the gel, as can be seen from the 3D reconstruction available in the Supporting Information (Video 2). In contrast, FF and FFmix hydrogels exhibit a different behavior. Cells appear to attach more readily to these hydrogels, as indicated by the elongated morphology of some cells (Supplementary Figure S13). Additionally, cell growth follows lines that correspond to the morphology of the gel itself (as compared to the electron microscopy images), suggesting a preferential proliferation onto the gel substrate (Video 1). Quantification of cell viability from the Live/Dead assay (Figure S14) further indicates that FF‐based hydrogels are more biocompatible than fF‐based ones. Likely the dimension of the pores of the hydrogels is crucial for cell viability, with large‐pore hydrogels favoring diffusion of nutrients or cell spreading and therefore cell survival.

Cell viability was measured using the MTT assay on NIH 3T3 fibroblasts using different concentrations of peptides (FF or fF) and mixtures (FFmix and fFmix). Results reported in the supplementary (Figure S15) suggest a certain toxicity of the starting materials at high concentrations.

Furthermore, cell death may also be associated with the low stability of the hydrogels in the cell culture medium; after 24 hours of incubation at 37 °C the lamellar structures that compose the gel start to disaggregate. Therefore, cells lack a support to grow and die (Supplementary Figure S16).

Beyond the inherent toxicity of the hydrogel precursors, the ability of cells to adhere and grow on the gels plays a crucial role in the final biocompatibility of the system. Hydrogels that facilitate easier cell attachment show higher viability compared to those where cell adhesion is limited.

## 3 Conclusions

Biomolecules are interesting scaffolds to produce biocompatible materials. In this work we demonstrated that homochiral and heterochiral peptides induce different secondary structures in assembled peptide‐PNA conjugates, which reasonably translate into a different supramolecular organization of the conjugates. Peptide‐PNA conjugates can be integrated into hydrogels formed by peptides; the chirality of the peptide determines the morphology of the assembled molecules and the properties of the hydrogels. Preliminary experiments show that the hydrogels support growth of NIH/3T3 cells; interestingly cell growth occurs only in specific directions, that is, along the nanotubes. The oriented cell growth driven by the hydrogel's structure presents an interesting opportunity for tissue engineering applications, particularly where a biodegradable scaffold is required. The mixture of homochiral conjugate FFat and the homochiral peptide FF yields a hydrogel with bigger pores as compared to the heterochiral mixture analyzed here. The dimension of the pores reasonably affects diffusion of nutrients within the gels and therefore the viability of cells cultured on these hydrogels, with large pores favoring cell growth. The instability of the hydrogels in cell culture media along with their toxicity limits the application of these hydrogels in tissue engineering at this stage.

## Supporting Information

Experimental procedures and characterization of the products are available.

## Conflict of Interest

The authors declare no conflict of interest.

## Supporting information



Supporting Information

Supporting Information

Supporting Information

## Data Availability

The data that support the findings of this study are available in the supplementary material of this article.
