# Supplementary material for: Self‐Assembly of Heterochiral and Homochiral Peptides Conjugated to PNA Dimers and Their Integration in Peptide‐Based Hydrogels
Source: Chemistry. 2025 Aug 13;31(49):e02255. doi: 10.1002/chem.202502255 (PMC12405829; doi:10.1002/chem.202502255)
Supplement: Supplementary file 1 — Supporting Information [file CHEM-31-e02255-s003.pdf]

Supporting information for

Self-assembly of heterochiral and homochiral peptides conjugated to PNA dimers and their integration in peptide based hydrogel

Ilaria Miglioli, Alessandro Ajò, Stefano Di Ciolo, Marco Carofiglio, Daria di Prisco, Dritan Siliqi, Luisa De Cola and Alessandra Romanelli

Synthesis of FFat, fFat and Ffat

The molecules were synthesized on solid phase on a Rink-Amide resin, following standard protocols.[1]

All compounds were purified by RP-HPLC on a Phenomenex Jupiter 10 μ Proteo 90 ˚A 100x21.2mm column and analyzed by analytical HPLC on a Sepachrom Vydamas 5μ C18 100 using a gradient of CH_3CN (0.1% TFA) in H_2O (0.1% TFA) in from 10 to 50% in 20 minutes. The identity of compounds was assessed by ESI-MS and NMR.
For all compounds the calculated mass is: 852.9.

NMR data are reported in tables S1 to S3. The spectra are appended at the end of the Supporting material (Figures S17-S34).

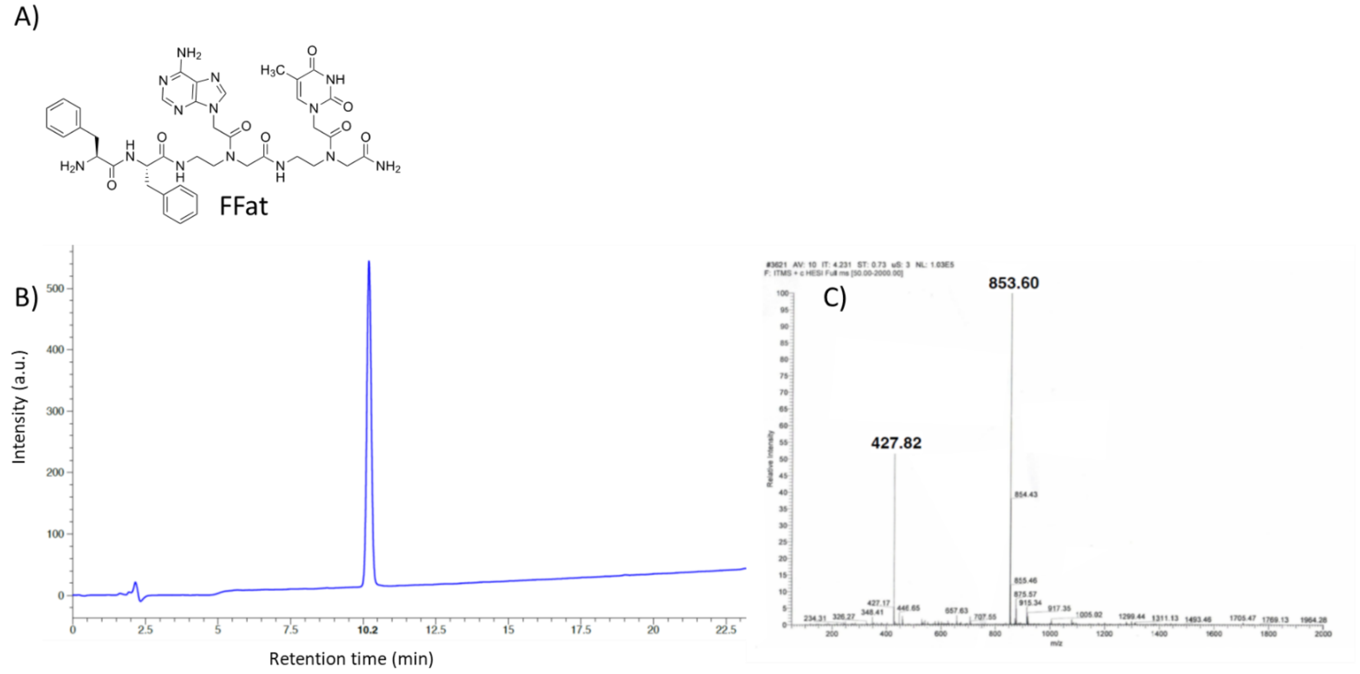

Figure S1: A) chemical structure of FFat; B) HPLC profile of the pure compound; C) ESI-MS of the compound.

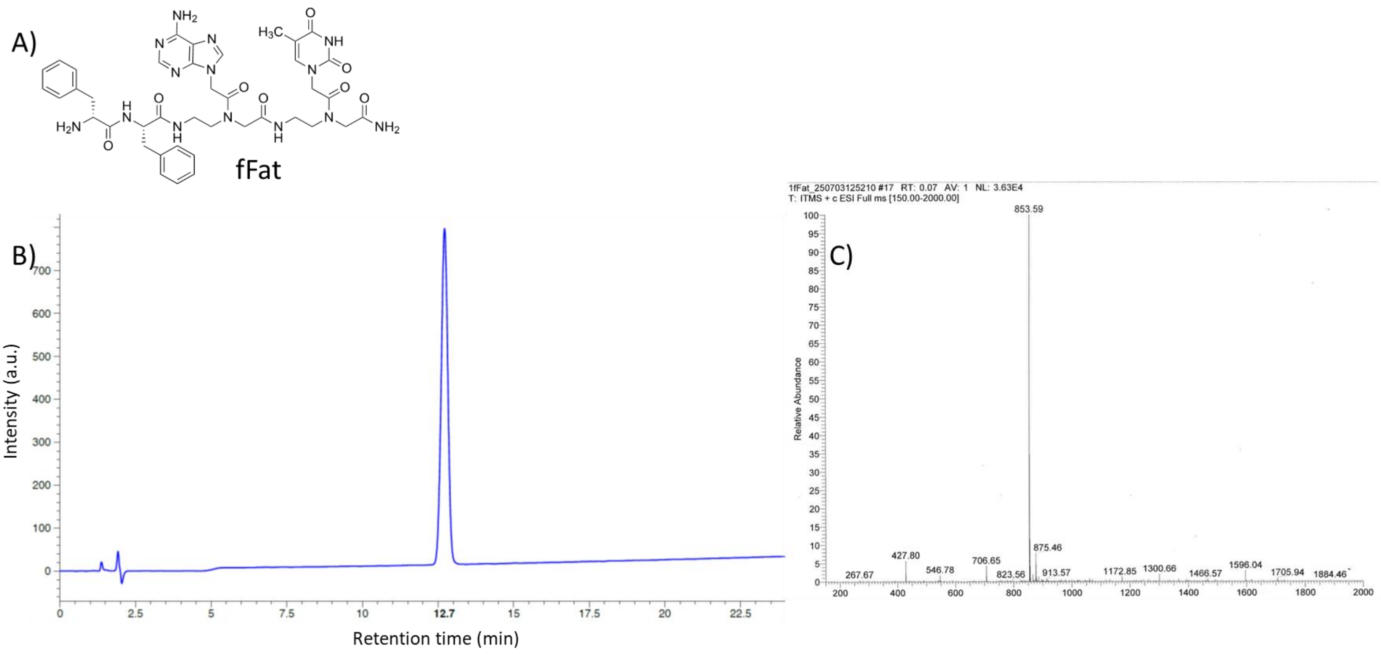

Figure S2: A) chemical structure of fFat; B) HPLC profile of the pure compound; C) ESI-MS of the compound.

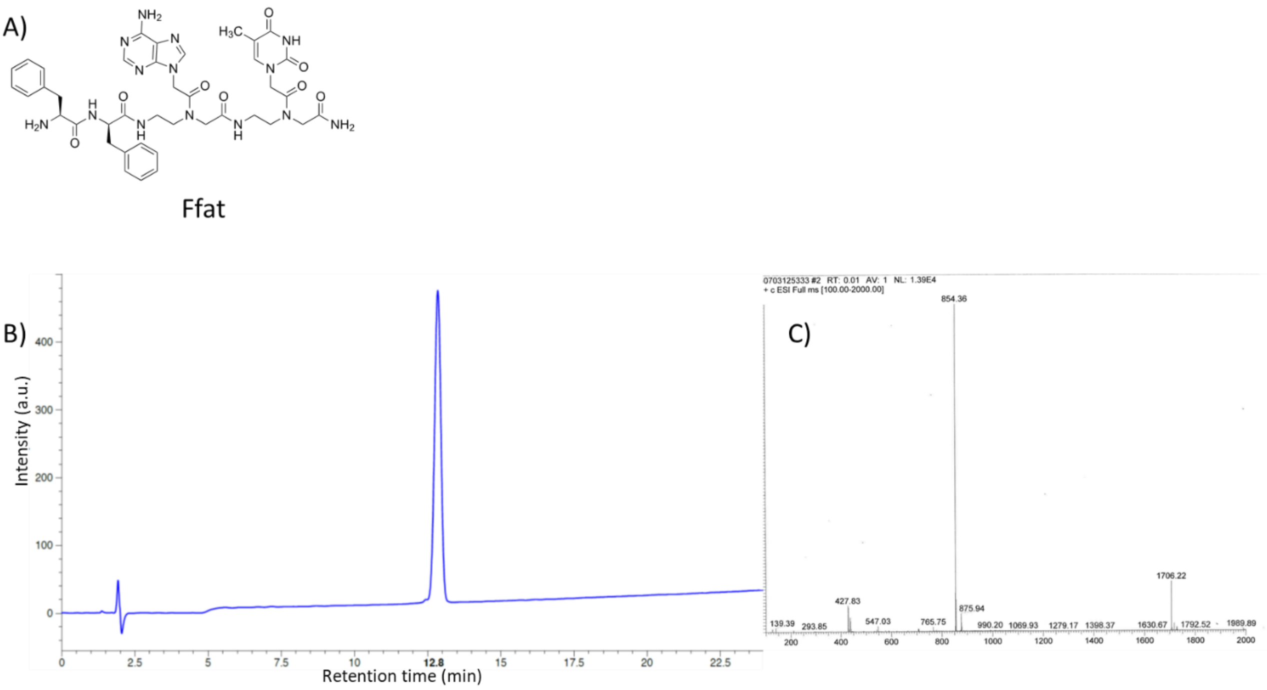

Figure S3: A) chemical structure of Ffat; B) HPLC profile of the pure compound; C) ESI-MS of the compound.

NMR data

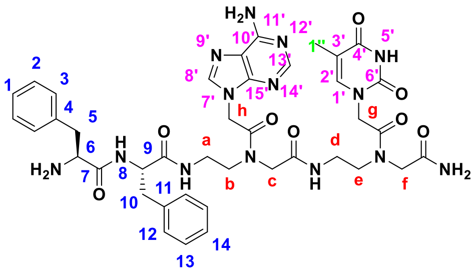

FFat

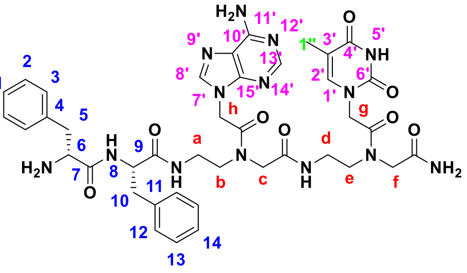

fFat

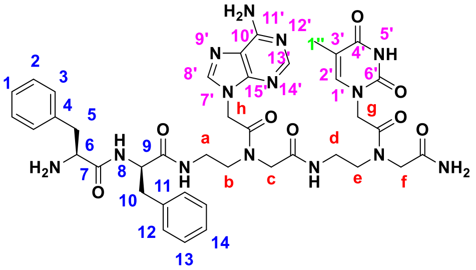

Ffat

NMR in DMSO-d^6: rotamers mixture

Table S1: NMR characterization of FFat

Atom number	^{1}H (400 MHz)		^{13}C (400 MHz)
	ppm	multeplicity	ppm
1	7.34-7.16	-	126.96/127.60
2	7.34-7.16	-	129.60/130.05
3	7.34-7.16	-	128.70/129.00
4			135
5	a)3.12-2.92<br>b)3.11-2.92	m<br>m	53.58
6	a)4.04<br>b)4.03	m<br>m	54.94
NH_2	a)8.05<br>b)8.01	-<br>-	
7			160-170
8 (NH)	a)8.86<br>b)8.80		
9	a)4.60<br>b)4.43	m<br>m	53.70
10	a)3.06-2.90<br>b)2.93-2.92		55.05
11			137
12	7.34-7.16	-	126.96/127.60
13	7.34-7.16	-	129.60/130.05
14	7.34-7.16	-	128.70/129.00
T(CH_3) 1’’	a)1.54<br>b)1.65	-<br>-	a)12.20<br>b)12.25

	c)1.70<br>d)1.73	-<br>-	c)12.29<br>d)12.33
T(CH) 2’	a)7.24<br>b)7.19<br>c)7.31<br>d)7.34	m<br>m<br>m<br>m	a)142.50<br>b)142.70
T(NH) 5’	a)11.21<br>b)11.24<br>c)11.19<br>d)11.18	s<br>s<br>s<br>s	
A(CH) 8’	a)8.14<br>b)8.17<br>c)8.18<br>d)8.19	s<br>s<br>s<br>s	144.03
A(CH) 13’	a)8.23<br>b)8.26<br>c)8.27	s<br>s	148.84
a	3.53-3.14	m	47.1
b	3.5-3.16	m	36.5-38.3
c	4.7-3.89	m	50.3
d	3.53-3.14	m	47.1
e	3.5-2.76	m	36.5-38.3
f	4.7-3.89	m	50.3
g	5.05-5.28	s	44.55-44.8
h	4.30-4.70	s	44.55-44.8
C 3’	-	-	108
C 6’	-	-	151
C 10’	-	-	118
C 15’	-	-	149
C 11’	-	-	158-159

Table S2: NMR characterization of Ffat

NMR in DMSO-d^6: rotamers mixture

Atom number	^{1}H (400 MHz)		^{13}C (400 MHz)
	ppm	multiplicity	ppm
1	7.37-6.90<br>(7.37-7.15 7.00-6.90)	-	126.96/127.60
2	7.37-6.90	-	129.60/130.05
3	7.37-6.90	-	128.70/129.00
4			135
5	a)2.83-2.62<br>b)2.82-2.60	m<br>m	53.58
6	a)4.05<br>b)4.04	m<br>m	54.94
NH_2	a)8.96<br>b)8.89	-<br>-	
7			160-170
8 (NH)	a-b)8.49		
9	a)4.51	m	53.70

	b)4.66	m	
10	a)2.95-2.66<br>b)3.06-2.76		55.05
11			137
12	7.37-6.90	-	126.96/127.60
13	7.37-6.90	-	129.60/130.05
14	7.37-6.90	-	128.70/129.00
T(CH_3) 1''	a)1.54<br>b)1.65<br>c)1.70<br>d)1.73	-<br>-<br>-<br>-	a)12.20<br>b)12.25<br>c)12.29<br>d)12.33
T(CH) 2'	a)7.24<br>b)7.19<br>c)7.31<br>d)7.34	m<br>m<br>m<br>m	a)142.50<br>b)142.70
T(NH) 5'	a)11.29<br>b)11.25<br>c)11.23<br>d)11.22	s<br>s<br>s<br>s	
A(CH) 8'	a)8.14<br>b)8.17<br>c)8.19<br>d)8.21	s<br>s<br>s<br>s	144.03
A(CH) 13'	a)8.25<br>b)8.28	s<br>s	148.84
a	3.53-3.14	m	47.1
b	3.5-3.16	m	36.5-38.3
c	4.7-3.89	m	50.3
d	3.53-3.14	m	47.1
e	3.5-2.76	m	36.5-38.3
f	4.7-3.89	m	50.3
g	5.05-5.28	s	44.55-44.8
h	4.30-4.70	s	44.55-44.8
C 3'	-	-	108
C 6'	-	-	151
C 10'	-	-	118
C 15'	-	-	149
C 11'	-	-	158-159

Table S3: NMR characterization of fFat

NMR in DMSO-d^6: rotamers mixture

Atom number	^1H (400 MHz)		^{13}C (400 MHz)
	ppm	multiplicity	ppm
1	7.37-6.90	-	126.96/127.60
2	7.37-6.90	-	129.60/130.05
3	7.37-6.90	-	128.70/129.00
4			135
5	a)2.83-2.62<br>b)2.82-2.60	m<br>m	53.58

6	a)4.05<br>b)4.04	m<br>m	54.94
NH_2	a)8.05<br>b)8.01	-<br>-	
7			160-170
8 (NH)	a)8.90<br>b)8.97		
9	a)4.51<br>b)4.66	m<br>m	53.70
10	a)2.95-2.66<br>b)3.06-2.76		55.05
11			137
12	7.37-6.90	-	126.96/127.60
13	7.37-6.90	-	129.60/130.05
14	7.37-6.90	-	128.70/129.00
T(CH_3) 1’’	a)1.54<br>b)1.65<br>c)1.70<br>d)1.73	-<br>-<br>-<br>-	a)12.20<br>b)12.25<br>c)12.29<br>d)12.33
T(CH) 2’	a)7.24<br>b)7.19<br>c)7.31<br>d)7.34	m<br>m<br>m<br>m	a)142.50<br>b)142.70
T(NH) 5’	a)11.29<br>b)11.25<br>c)11.23<br>d)11.22	s<br>s<br>s<br>s	
A(CH) 8’	a)8.14<br>b)8.17<br>c)8.19<br>d)8.21	s<br>s<br>s<br>s	144.03
A(CH) 13’	a)8.25<br>b)8.28	s<br>s	148.84
a	3.53-3.14	m	47.1
b	3.5-3.16	m	36.5-38.3
c	4.7-3.89	m	50.3
d	3.53-3.14	m	47.1
e	3.5-2.76	m	36.5-38.3
f	4.7-3.89	m	50.3
g	5.05-5.28	s	44.55-44.8
h	4.30-4.70	s	44.55-44.8
C 3’	-	-	108
C 6’	-	-	151
C 10’	-	-	118
C 15’	-	-	149
C 11’	-	-	158-159

Fluorescence measurements

The experiments were conducted on a spectrofluorometer Fluorolog Jobin Yvon Horiba using 1 cm path length cuvette.

For the determination of the CAC, increasing concentration of the compounds were added to a ANS solution 0.2 M in water.[2] Excitation wavelength was set at 350 nm, emission at 490 nm was plotted vs concentration, see Figure S4. In addition, concentration of the sample was plotted against the fluorescence emission intensity at 310 or 320 nm, see Figure S5.

The emission spectra were registered exciting at different wavelengths in a range between 310-360 nm; the excitation spectra were acquired at different emission wavelengths from 390 to 431 nm. To detect REES, spectra were recorded exciting between 310 and 500 nm, see Figure S6. Samples were dissolved in phosphate buffer 0.1M at pH 7.4 at a concentration 10x CAC. Excitation and emission spectra were acquired using the same slits parameters, each product needed different slits. The data plotting was performed by OriginLab software.

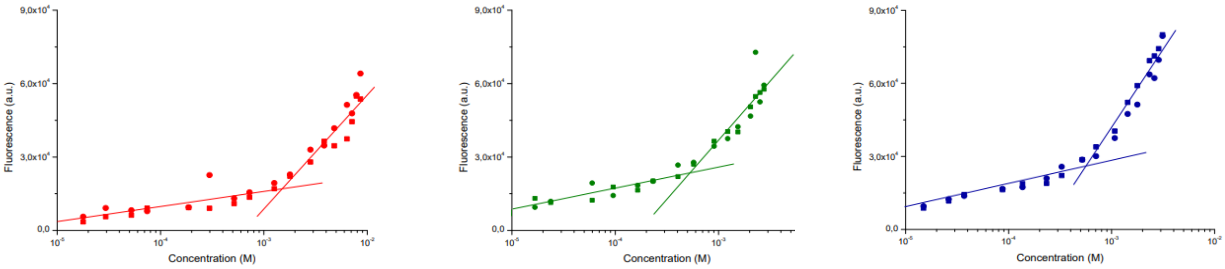

(a) FFat (b) Ffat (c) fFat

Figure S4: CAC determination. Plot of the fluorescence intensity at 490 nm of ANS solutions with increasing concentration of peptide-PNA conjugates vs peptide-PNA conjugate concentration.

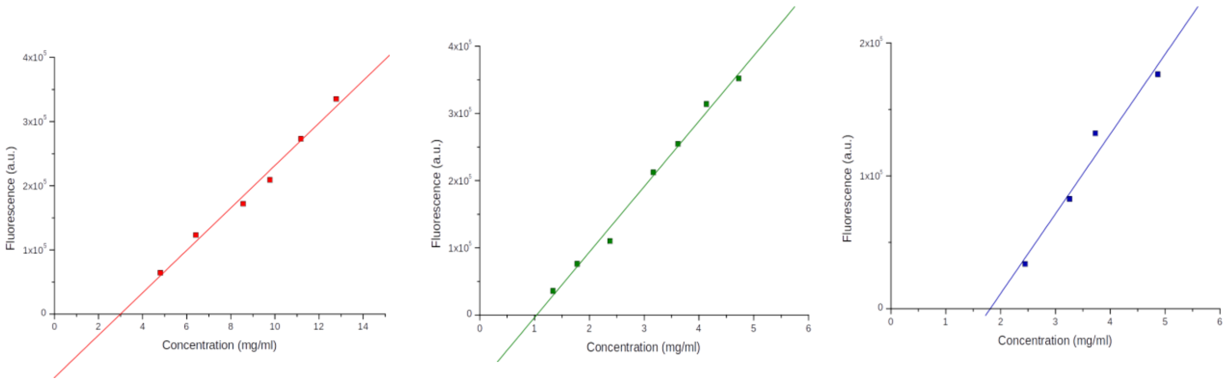

Ex 320 Em 410 Ex 330 Em 392 Ex 310 Em 410

Figure S5 CAC determination: fluorescence emission at 410/390 nm of FFat, fFat and Ffat (from left to right)

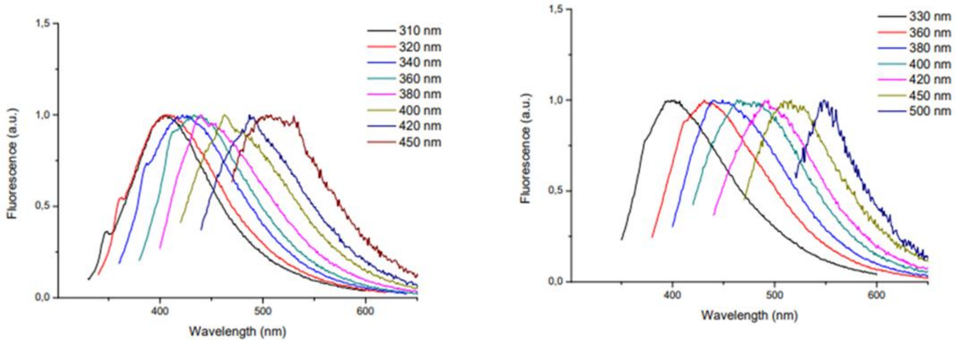

(a) FFat (b) Ffat

Figure S6: normalized fluorescence spectra recorded at different wavelengths.

Circular Dichroism

The circular dichroism spectra were recorded using a Jasco J-815 spectropolarimeter (Jasco, Easton, MD) at 25 ºC from 190 nm to 320 nm (1 nm bandwidth and 0.1 nm resolution). The measurements were executed in a 0.1 mm optical path quartz capillary cuvette at 25°C in phosphate buffer 0.1 M pH 7.4 at a sample concentration 5x CAC. CD spectra are reported in molar ellipticity and measured in units of mdeg as a function of wavelength.

Hydrogel preparation

Peptides and peptide-PNA conjugates are suspended in phosphate buffer 0.1 M, pH 7.4 in different ratios. The suspension is warmed in an oil bath to 90°C and then it is slowly cooled down to room temperature. Incorporation of the PNA-peptide in the hydrogel is demonstrated by the HPLC analysis of the hydrogel after it was broken by addition of water and acetonitrile and vortexed to give a solution, that was diluted and analyzed by RP-HPLC. As an example we show the HPLC profiles obtained after analysis of one of the hydrogels formed by the heterochiral mixtures (Figure S7).

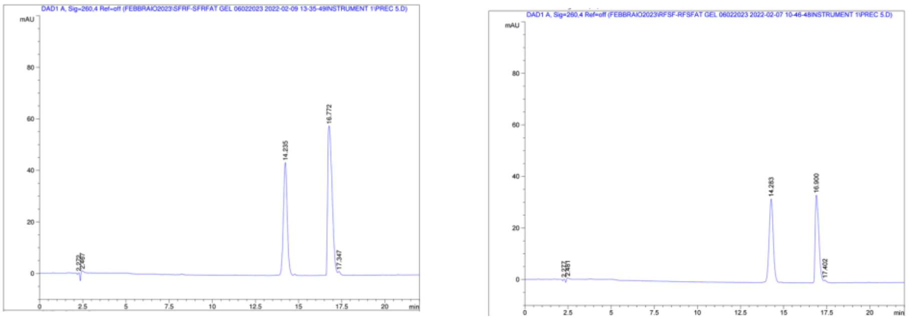

Figure S7: RP-HPLC of the hydrogels composed of Ff (1.3%w/v) +Ffat (0.07% w/v) (left) and fF (1.3%w/v) +fFat (0.07% w/v) (right) followed at 260nm. The analysis was carried out using a gradient of acetonitrile (0.1% TFA) in water (0.1% TFA) from 5 to 50 % in 20 minutes. The methos was optimized to separate the PNA-peptide and the peptide. The peak at lower retention time corresponds to the PNA-peptide conjugate, the peak at the higher retention time corresponds to the peptide.

Hydrogels composed of peptides are named using the name of the peptide, gels formed by the mixture of peptide and PNA-peptide conjugated are named using the name of the peptide, followed by “mix”.

Fluorescence of hydrogels

The hydrogels (1.5 % w/v of peptide or 1.5 % w/v of peptide+ 0.2% w/v of peptide-PNA conjugates) were placed into a quartz tube; fluorescence spectra are reported in Figure S8.

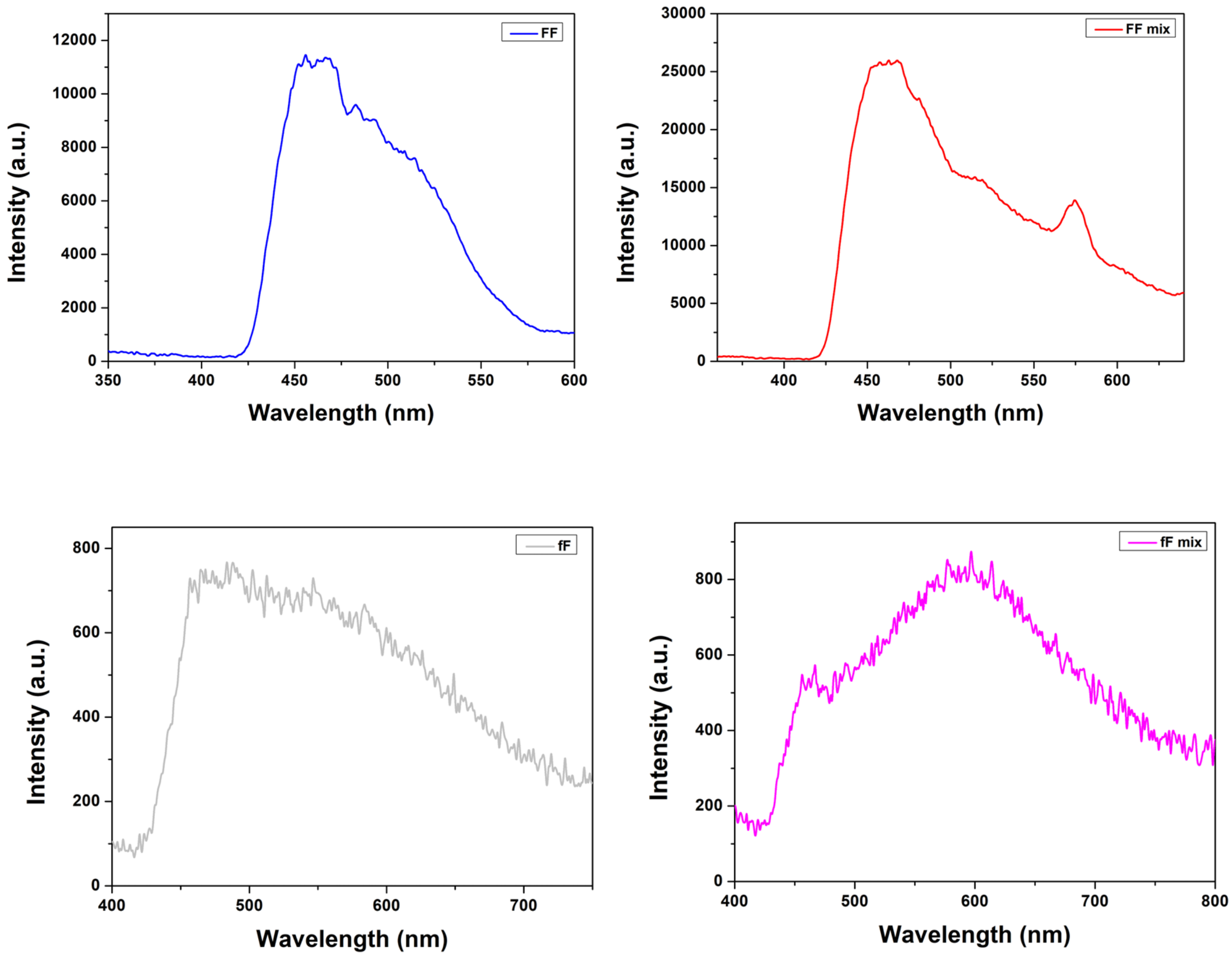

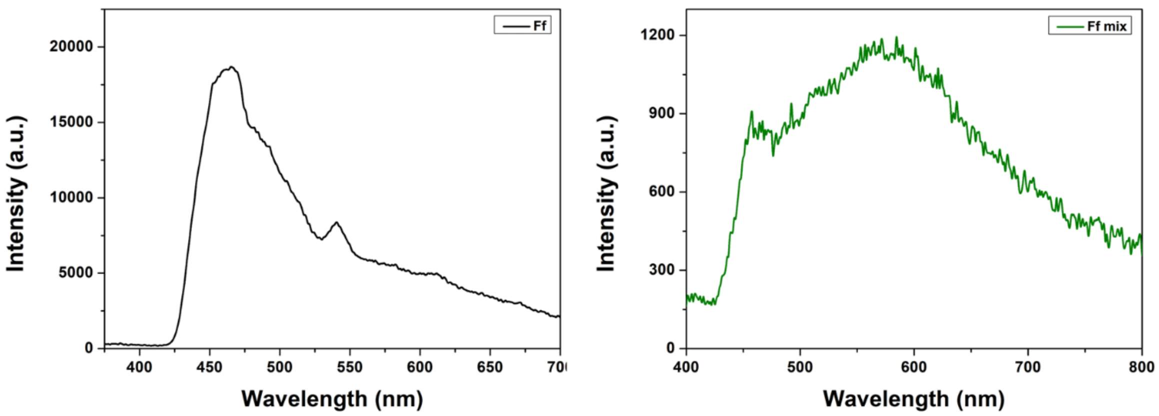

Figure S8: Fluorescence of the lyophilized hydrogels indicated in each panel using the following excitation wavelength: FF λ_{exc} 320 nm; FFmix λ_{exc} 340 nm; Ff λ_{exc} 330 nm; Ffmix λ_{exc} 360 nm; fF λ_{exc} 310 nm; fFmix λ_{exc} 360 nm.

XRD measurements

Below are reported all hydrogels analyzed by X-ray powder diffraction techniques at the XRD1 DH Phaser (Bruker), in transmission mode, at room temperature on lyophilized hydrogel composed of 1.5 % w/v of peptide or 1.5 % w/v of peptide+ 0.2% w/v of peptide-PNA conjugates. The hydrogel powder was loaded in a Teflon holder for the measurement. The scan type used is coupled two theta/theta with continuous PSD fast scan mode. The measurements start at 10 ° and end at 60 ° with a step size of 0.008 °, a total of 6150 steps were performed. The diffraction pattern shows the crystalline structure of the hydrogels. There are no significant differences between the patterns. We can assume that the supramolecular architecture of gels is formed by microcrystals assembled.[3] An XRD analysis was performed on only Teflon holder to be sure that peaks are coming from sample and not from the sample holder.

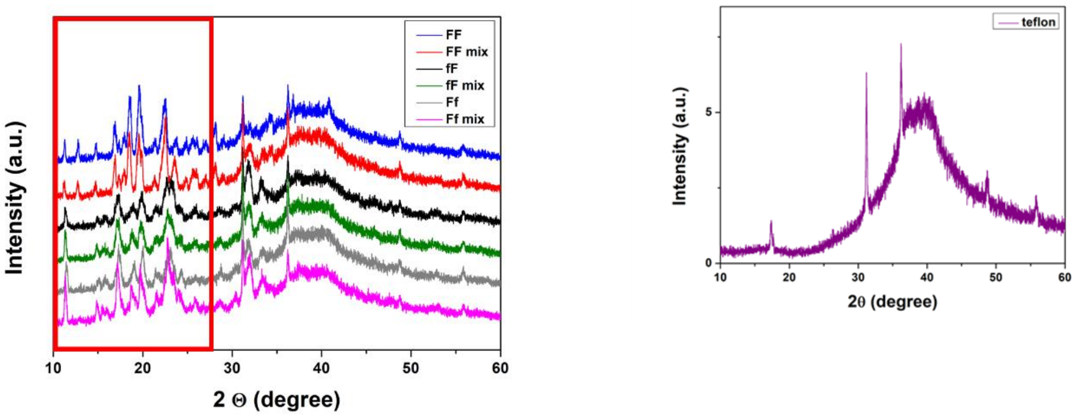

Figure S9. XRD analysis of lyophilized hydrogels

Scanning electron microscopy experiments

The analyses were performed on lyophilized hydrogels, composed of 1.5 % w/v of peptide or 1.5 % w/v of peptide+ 0.2% w/v of peptide-PNA conjugates.

Scanning electron microscopy was conducted using FE – SEM Sigma (Zeiss) using inlens detector (2 KV and 3.5 WD).

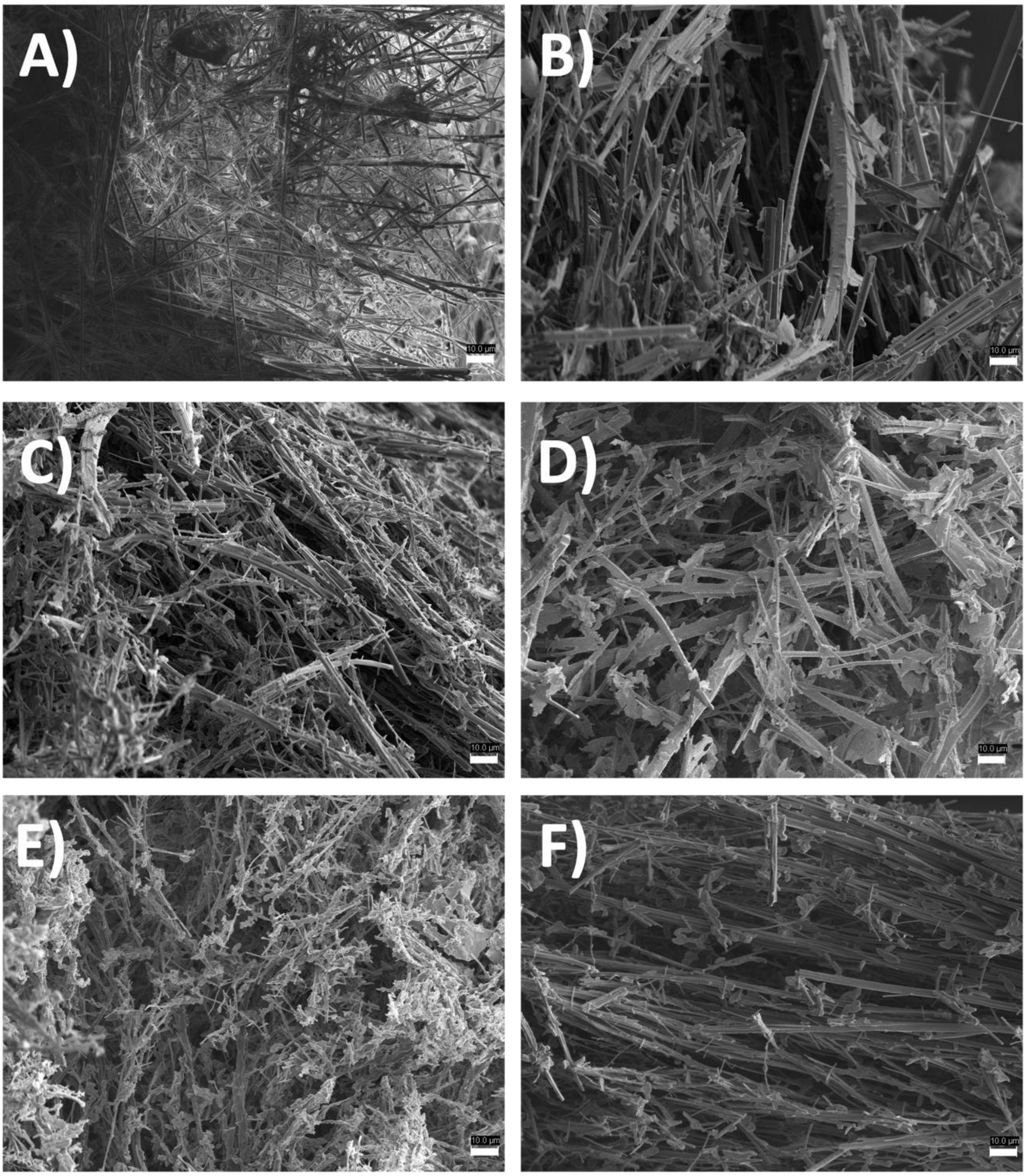

Figure S10: SEM images of A) FF ; B) FFmix ; C) fF; D) fFmix ; E) Ff and F)Ffmix The scale bar reported in the picture is 10 µm.

SAXS measurements

The small-angle X-ray scattering (SAXS) setup includes the SAXSess mc2 instrument from Anton Paar GmbH (Graz, Austria), containing a slit collimator system, and the PW3830 laboratory X-ray generator (40 kV, 50 mA) with a long-fine focus sealed X-ray tube (CuKα λ = 0.1542 nm) from PAN analytical. Detection was performed with the 2D imaging-plate reader Cyclone® by Perkin Elmer, placed at 0.8 m from the sample. The 2D data were converted to 1D data and back-ground corrected by using SAXSQuant software (Anton Paar

GmbH). Measurements were performed on hydrogels as well as on lyophilized hydrogel samples for 15 min. Samples analyzed include hydrogels composed of 1.5 % w/v of peptide or 1.5 % w/v of peptide + 0.2% w/v of peptide-PNA conjugates. The hydrogel samples were placed into the metal sample holder with apertures and the measure was performed under vacuum to minimize air scattering. The lyophilized hydrogels were finely ground into powder and placed into the metal sample holder with apertures and again the measure was performed under vacuum (Figure S11).

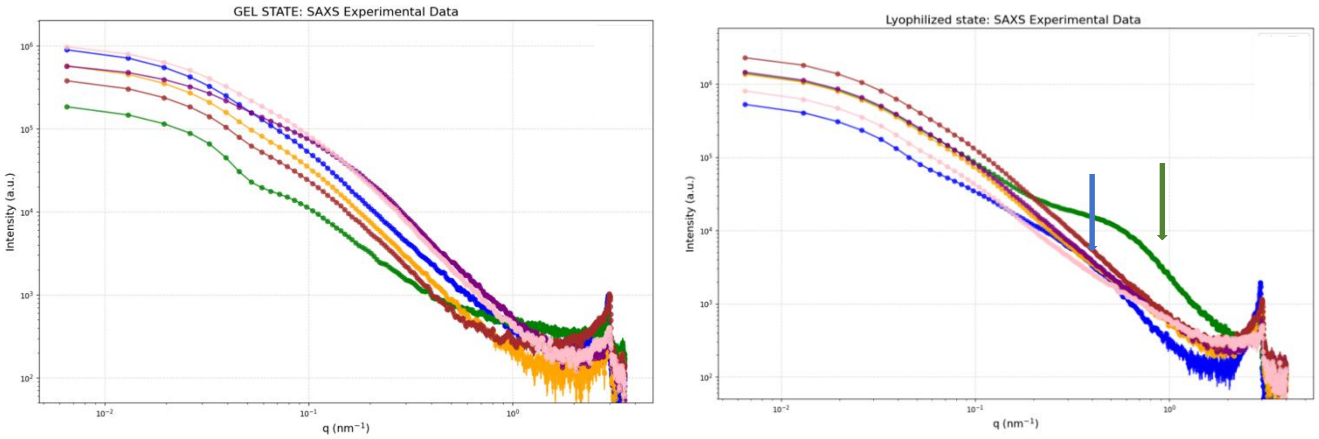

Figure S11: SAXS experimental profiles on gel state (panel on the left) and lyophilized hydrogels (panel on the right). Blue: FF, green: FFmix, yellow: Ff, purple: Ffmix, brown: fF, pink: fFmix.

For both states (gel and lyophilized) two modelling fitting approach were used: i) gel model ii) cylindrical and/or ellipsoid shape models. Tables S4, S5, S6 and S7 summarize the refined parameters for the hydrogel and lyophilized hydrogels. The fitting is represented in Figure 7 for the hydrogels (main text) and in Figure S12 for the lyophilized hydrogels.

Criteria Used (arbitrarily chosen):

1. Pore Size – based on Correlation Length (ξ)

   < 20 nm → small pores

   20–35 nm → medium pores

   > 35 nm → large pores

2. Connectivity – based on Fractal Dimension (Df)

   < 2.1 → low connectivity

   2.1–2.5 → moderate connectivity

   > 2.5 → high connectivity

3. Regularity – based on χ^2 and Df proximity to 2.5–2.8

   $\chi^2 < 2$ and Df ≈ 2.5–2.8 → regular

$\chi^2 > 4$ or Df far from 2.5 → irregular

Others → moderately regular

4. Structural Extent (Compactness) – based on Rg

< 15 nm → compact structure

15–30 nm → moderately extended

> 30 nm → highly extended

Table S4: Gel state: gel model fitting data

Sample	**Rg** (nm)	ξ (nm)	**Df**	χ^2	***Key Structural Features***
FF	37.0	31.7	2.4	0.7	medium pores, moderate connectivity, moderately regular, highly extended
FFmix	30.3	37.0	2.2	1.4	large pores, moderate connectivity, moderately regular, highly extended
Ff	12.0	39.3	2.2	1.8	large pores, moderate connectivity, moderately regular, compact
Ffmix	8.4	29.9	2.0	1.7	medium pores, low connectivity, irregular, compact
fF	62.9	10.3	2.9	1.1	small pores, high connectivity, irregular, highly extended
fFmix	28.9	22.0	2.6	1.6	medium pores, high connectivity, regular, moderately extended

Table S5: Gel state: a mixed model (combination of ellipsoid and cylinder components) fitting data

Sample	Ellipsoid			***Cylinder***			
	**w (%)**	**Rp** (nm)	$\mathbf{R_{eq}}$(nm)	***w(%)***	***R(nm)***	***H (nm)***	χ^2
FF	36.0	4.7	29.0	64.0	77.5	1.66	2.0
FFmix	26.0	1.9	2830.0	74.0	8.0	14111	29.4
Ff	40.0	9.6	65.0	60.0	5.0	8886	15
Ffmix	27.0	17.6	79.2	73.0	16.3	3.6	3.1
fF	77.0	3.4	15.7	23.0	21.1	1122	5.9
fFmix	47.0	166.2	18.0	53.0	10.9	4.1	3.1

Table S6: Lyophilized state: gel model fitting data

Sample	**Rg** (nm)	ξ (nm)	**Df**	χ^2	***Key Structural Features***
FF	5.32	49.59	1.9	2.1	large pores, low connectivity, irregular, compact
FFmix	2.63	33.84	2.32	8.8	medium pores, moderate connectivity, irregular, compact
Ff	41.46	37.00	2.25	1.4	large pores, moderate connectivity, moderately regular, highly extended

Ffmix	13.99	43.49	2.10	1.9	large pores, moderate connectivity, moderately regular, compact
fF	12.00	38.14	2.21	4.9	large pores, moderate connectivity, irregular, compact
fFmix	16.86	52.49	1.93	0.6	large pores, low connectivity, irregular, moderately extended

Table S7: Lyophilized state: a mixed model (combination of ellipsoid and cylinder components) fitting data

Sample	Ellipsoid			***Cylinder***			
	**%**	**R_p** (nm)	**R_{eq}**(nm)	***%***	***R(nm)***	***L (nm)***	χ^2
FF	52	0.63	72.29	48	3.84	6223.9	4.0
FFmix	24	243.2	16.9	76	3.9	2.7	20.9
Ff	39	25.00	89.24	61	22.0	3.0	6.2
Ffmix	44	6.28	57.52	56	2.56	1654.7	23.1
fF	100	2.21	65.22	0	-	-	82.1
fFmix	14	32.1	92.2	86	22.6	0.5	1.5

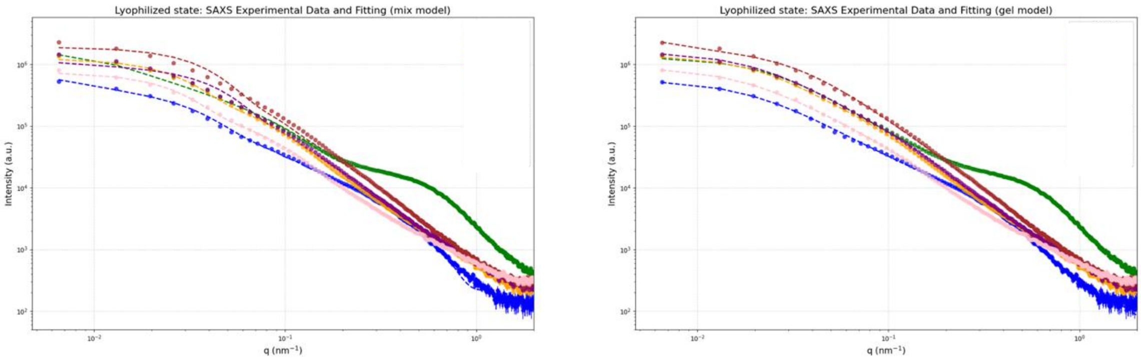

Supplementary Figure S12: Lyophilized state analysis with a mixed model (combination of ellipsoid and cylinder components) (panel on the left) and a gel model (panel on the right). Both models are represented by dashed lines and are fitted to the experimental data, which are shown as filled circle symbols. Blue:FF, green: FFmix, yellow: Ff, purple: Ffmix, brown: fF, pink: fFmix.

Cell growth on hydrogel

To evaluate the biocompatibility of the hydrogels formulated as described in the previous sections, a Live/Dead assay was conducted using a fibroblast cell line. The fibroblast NIH/3T3 (ATCC, CRL-1658) cell line was selected as representative cell line to determine the behavior of the hydrogels in a biological environment.

The hydrogels (fF, fF-peptide, FF and FF-peptide) were directly cast into the wells of a 96-well plate (TC-treated, Euroclone). After cooling to room temperature, 4 x 10^4 cells per well were seeded onto the gels, along with 100 µl of cell culture medium (DMEM, Gibco, supplemented with 10% of Fetal Bovine Serum (FBS, Gibco), 1% of L-Glutamine (Euroclone) and 1% of Penicillin-Streptomycin (Euroclone, 100 µg/mL of streptomycin and 100 units/mL of penicillin). Cells were incubated at 37 °C in a 5 % CO2 atmosphere for 24 hours to allow their attachment to the gel.

Following incubation, the culture medium was replaced by medium containing Live/Dead staining reagents (ThermoFisher). Specifically, Calcein AM was added to obtain a final concentration of 2 µM, and Ethidium homodimer-1 to a final concentration of 4 µM. Cells were incubated for 30 minutes at 37°C, then imaged using a Nikon Ti2 Eclipse A1 laser confocal microscope. The images were analyzed using NIS-Element software (Nikon), with confocal imaging providing detailed information on the three-dimensional spatial distribution of the cells.

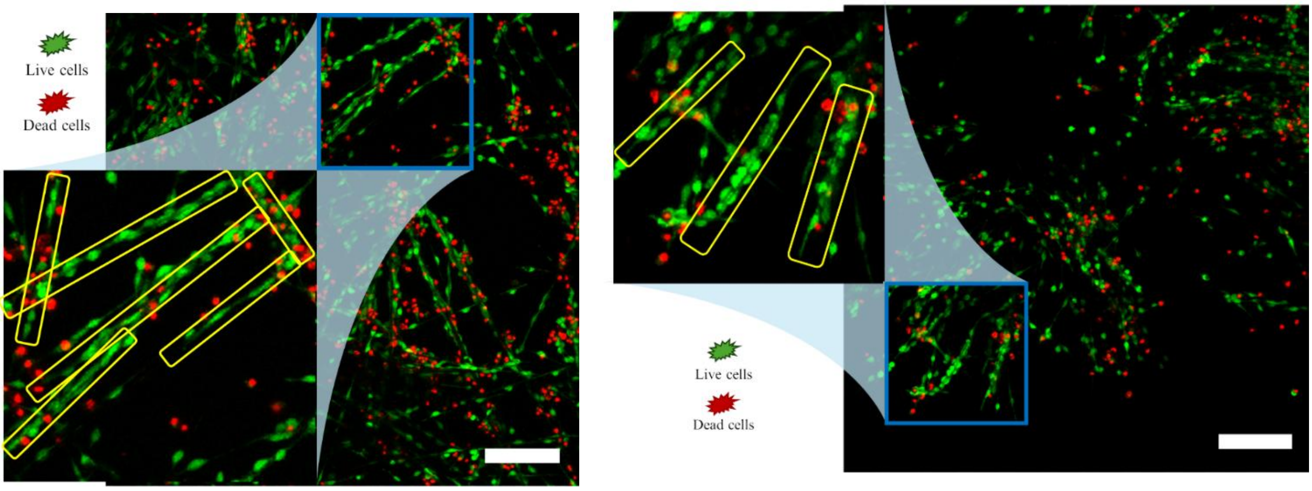

Figure S13: left**:** FF hydrogel image with a zoomed region to show elongated cell morphology; Right: FFmix hydrogel, with a zoomed region to show elongated cell morphology

To quantify cell viability, live and dead cells were counted in four different samples using ImageJ software. Viability was calculated as the percentage of live cells relative to the total cell population.

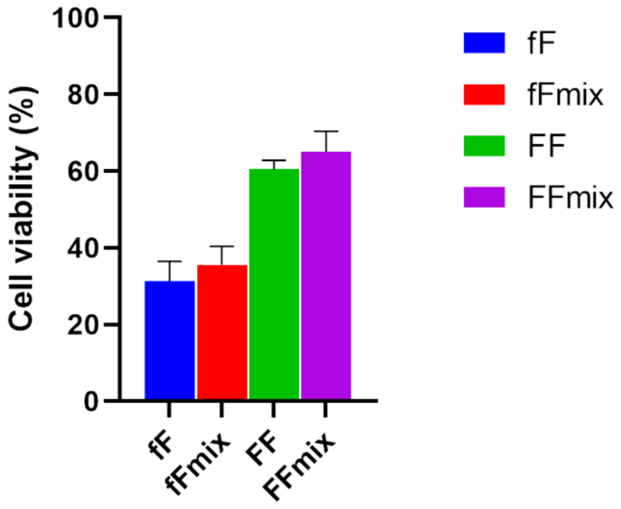

Figure S14: Viability quantification of NIH/3T3 cells through Live/Dead stained fluorescence microscopy images.

MTT assay

Cell viability was measured using the MTT assay, which is based on the conversion of MTT to formazan crystals by mitochondrial dehydrogenases. Briefly, NIH 3T3 (fibroblasts) cells were seeded into the 96-well plates at a density of $4x10^4$ cells/mL, in a volume of 100 µL of supplemented culture media (DMEM, supplemented with L-Glut, P/S, FBS). After 24h, cells were treated with different concentrations (12,5-25-50mM) of FF (Figure 15 a), FFat (figure 15-b), fF (figure 15-c), fFat (figure 15-d) and incubated at 37 °C for 24 h. The medium was removed and 1:10 MTT/medium culture solution was added to the wells. Plates were incubated at 37 °C for 3h. Subsequently, the MTT solution was removed and the obtained formazan violet product was dissolved in 100 µL of Isopropanol with HCl 0.04 M. Absorbance was measured using a microplate reader (Tecan, The Infinite 200 PRO) at 565 nm. All readings were compared with the control, which represented 100% viability. Cell viability was calculated as follow % $=\frac{Abs\ sample}{Abs\ control}$ x 100.

Each experiment was performed in biological triplicate.

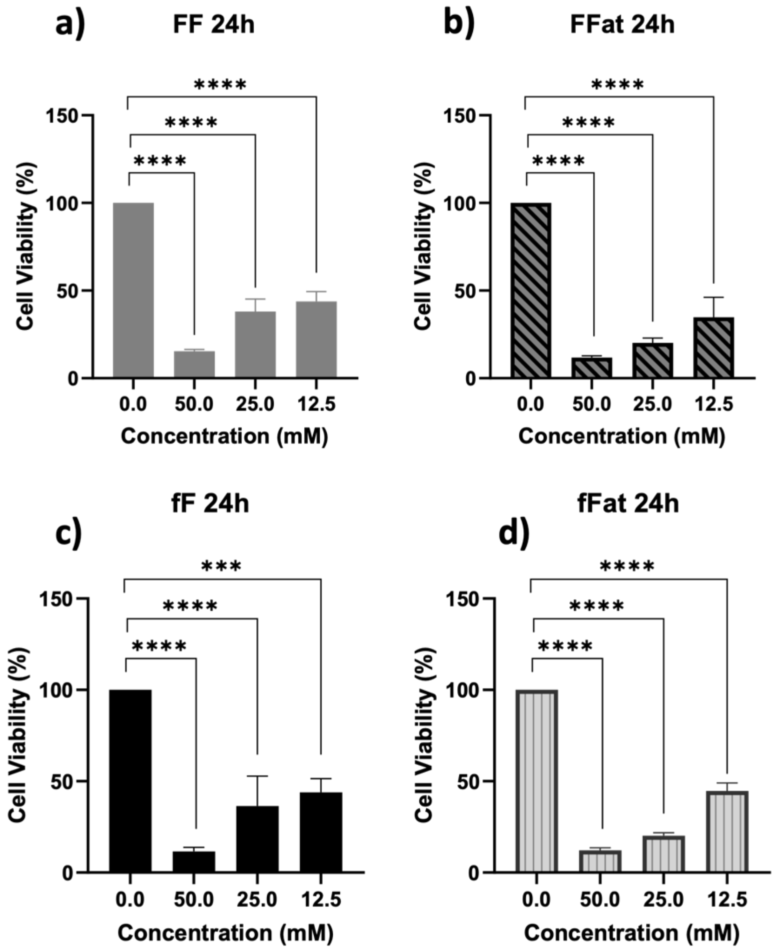

Figure S15: cell viability of: FF at different concentration, from 50mM to 12.5mM a); FFat at different concentrations, from 50mM to 12.5mM b); fF at different concentrations, from 50mM to 12.5mM c); fFat at different concentrations from 50mM to 12.5mM d). Each experiment was performed in biological triplicate and the results are expressed as mean ± standard. One-way ANOVA test was used for statistical analysis. ***p≤0.001, ****p≤0.0001.

Comparing the different concentrations, we can observe that at high dose concentration there is an increase of toxicity resulting in a low viability. The concentration with approximately 50% viability is the 12.5mM peptide administered.

Degradation experiment

Hydrogels were prepared as described in "Cell growth hydrogel". Images were taken before staining with brightfield microscopy using an Olympus IX71 Inverted Research Microscope in order to determine the stability of the material and subsequential degradation in lamellar structures. The hydrogels were prepared

using the same experimental conditions of cell growing experiment in cell culture medium (DMEM, supplemented with L-Glut, P/S, FBS) for 24h in static incubator at 37C. The images (sample FF reported in the pictures below) were then captured before the staining for confocal experiment. As we can see from the microscopy pictures below the hydrogel degrade completely and all the lamellar that compose the gel start to disaggregate.

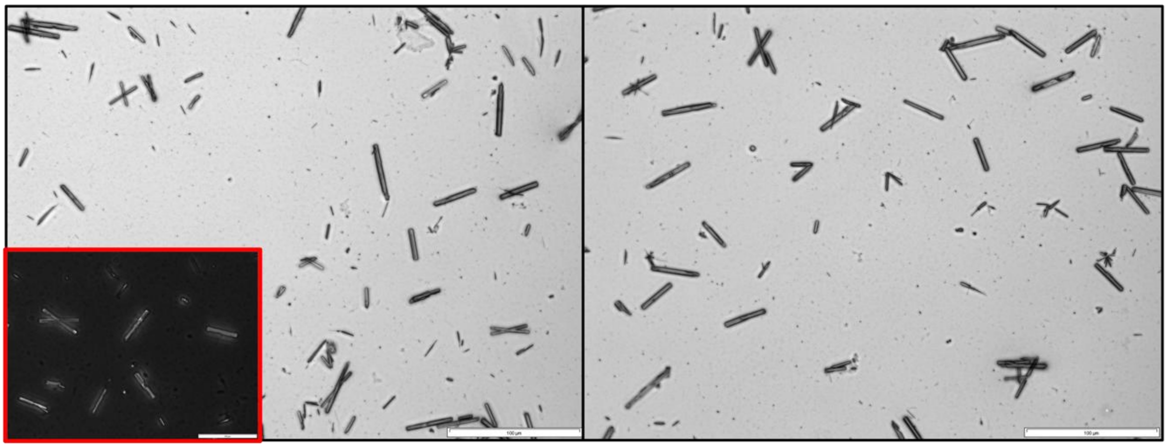

Figure S16: images of hydrogel FF after 24h incubation in the cell culture media at 37°C.

NMR spectra

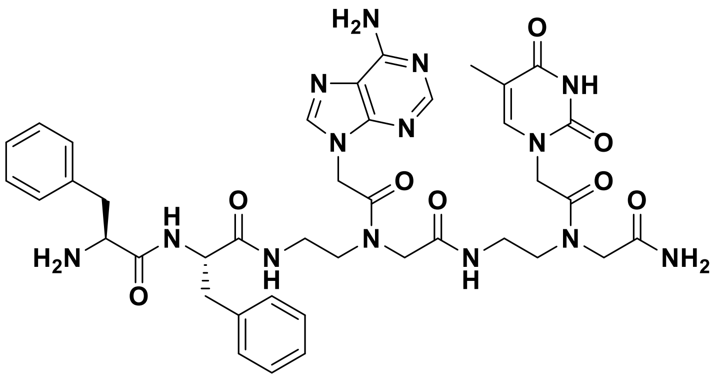

FFat

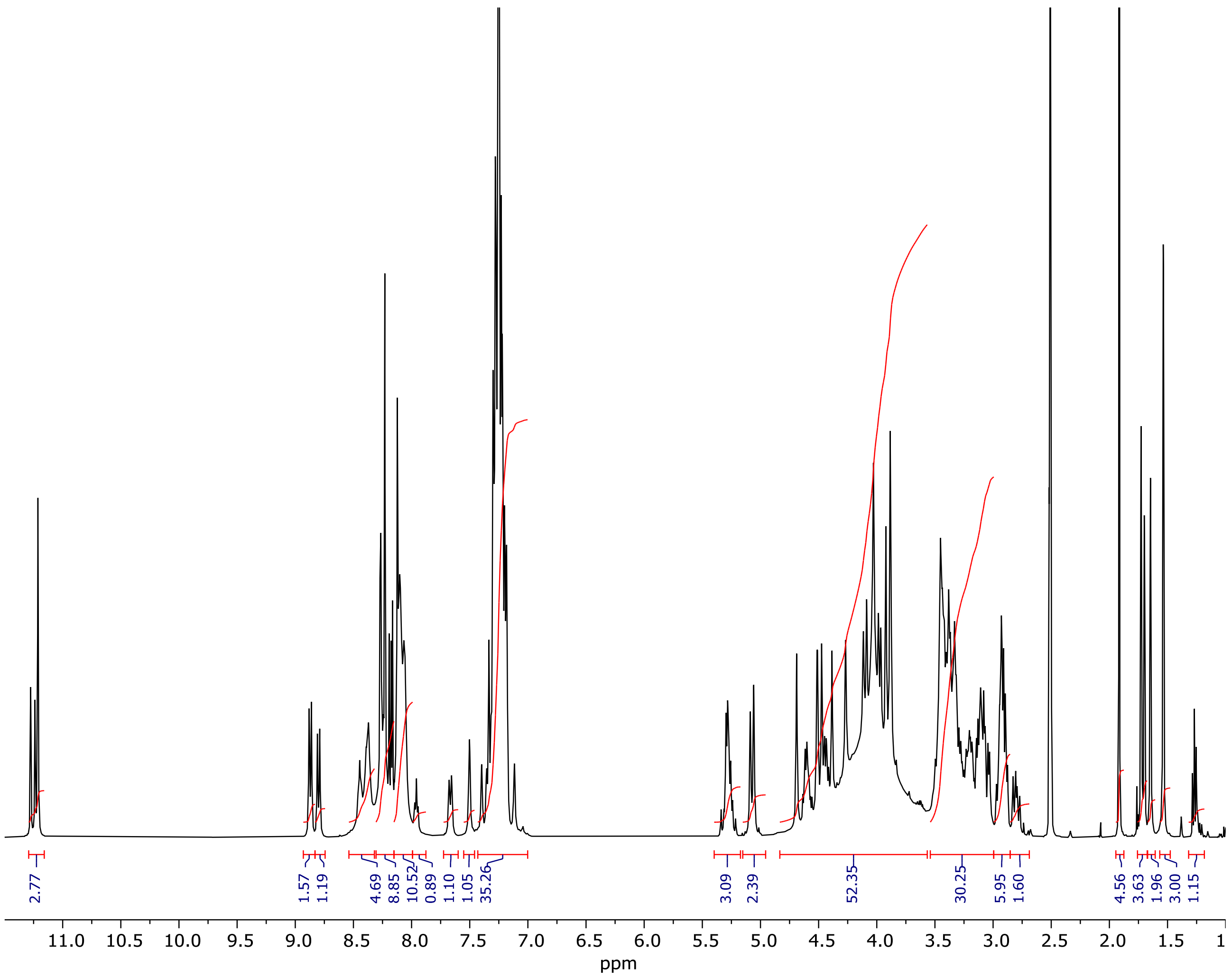

Figure S17: The chemical structure of the molecule and ^{1}H NMR (400 MHz, DMSO, T = 300K) of FFat**.**

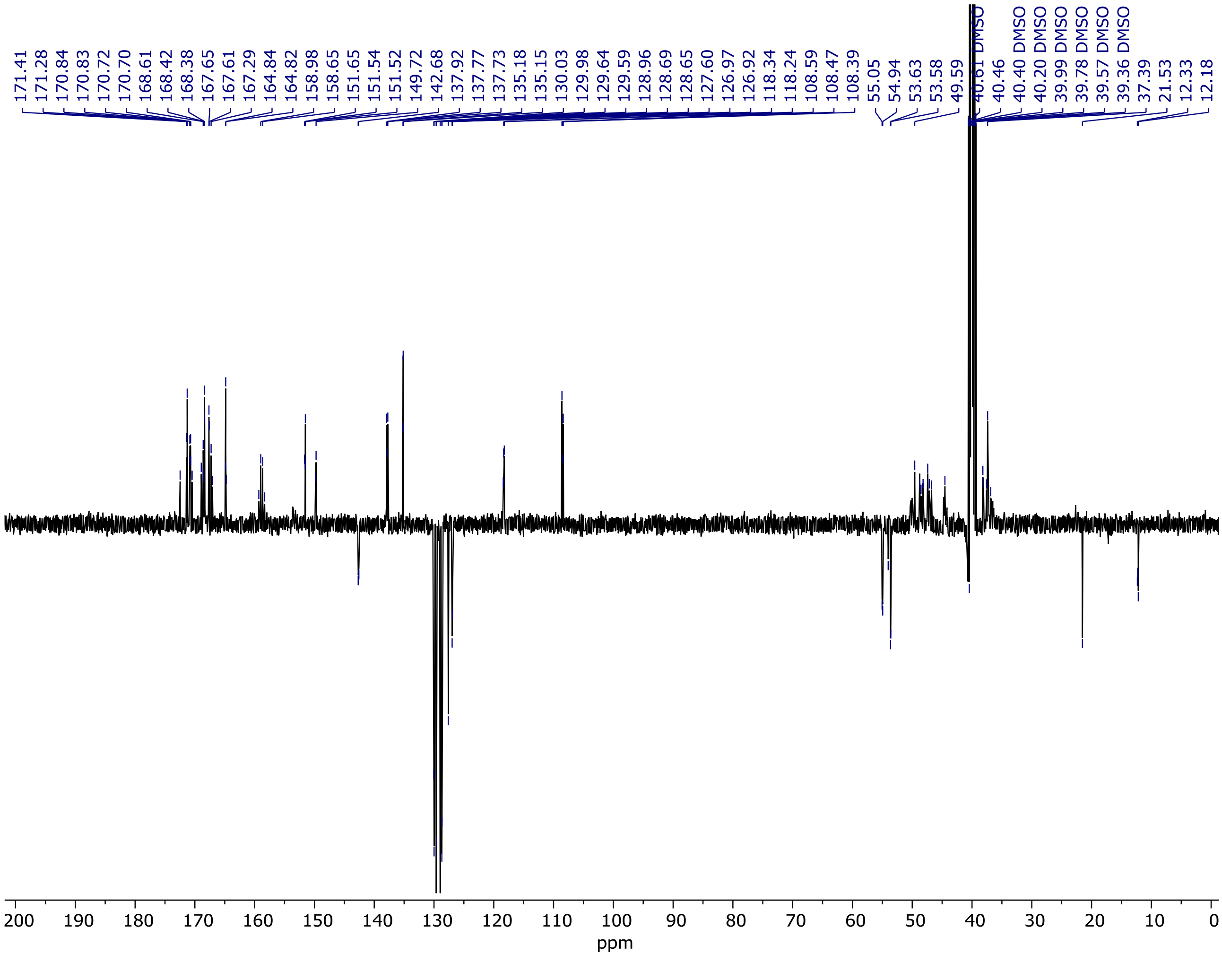

Figure S18: ^{13}C NMR-apt (100 MHz, DMSO, T = 300K) of Ffat.

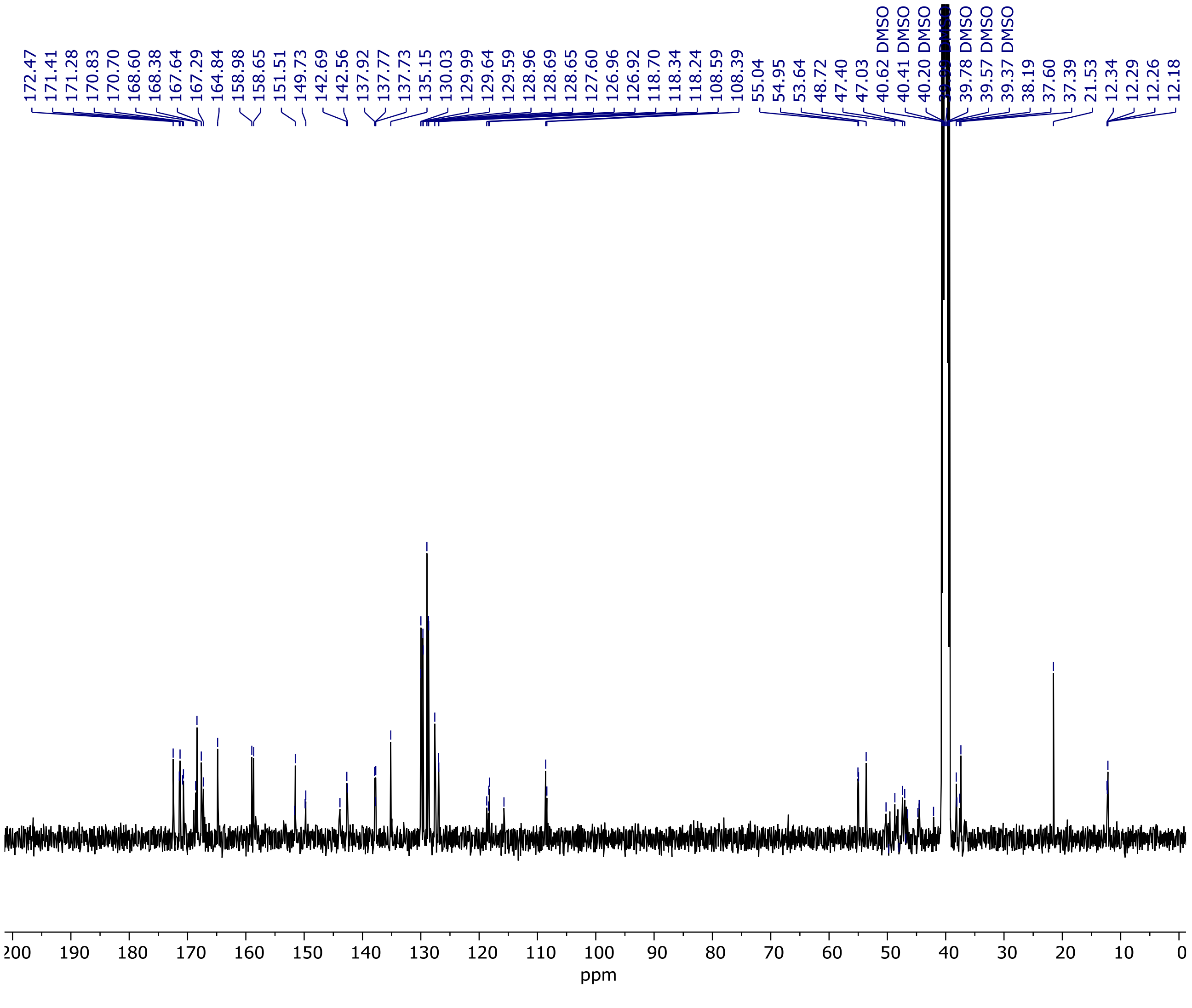

Figure S19: ^{13}C NMR (100 MHz, DMSO, T = 300K) of Ffat.

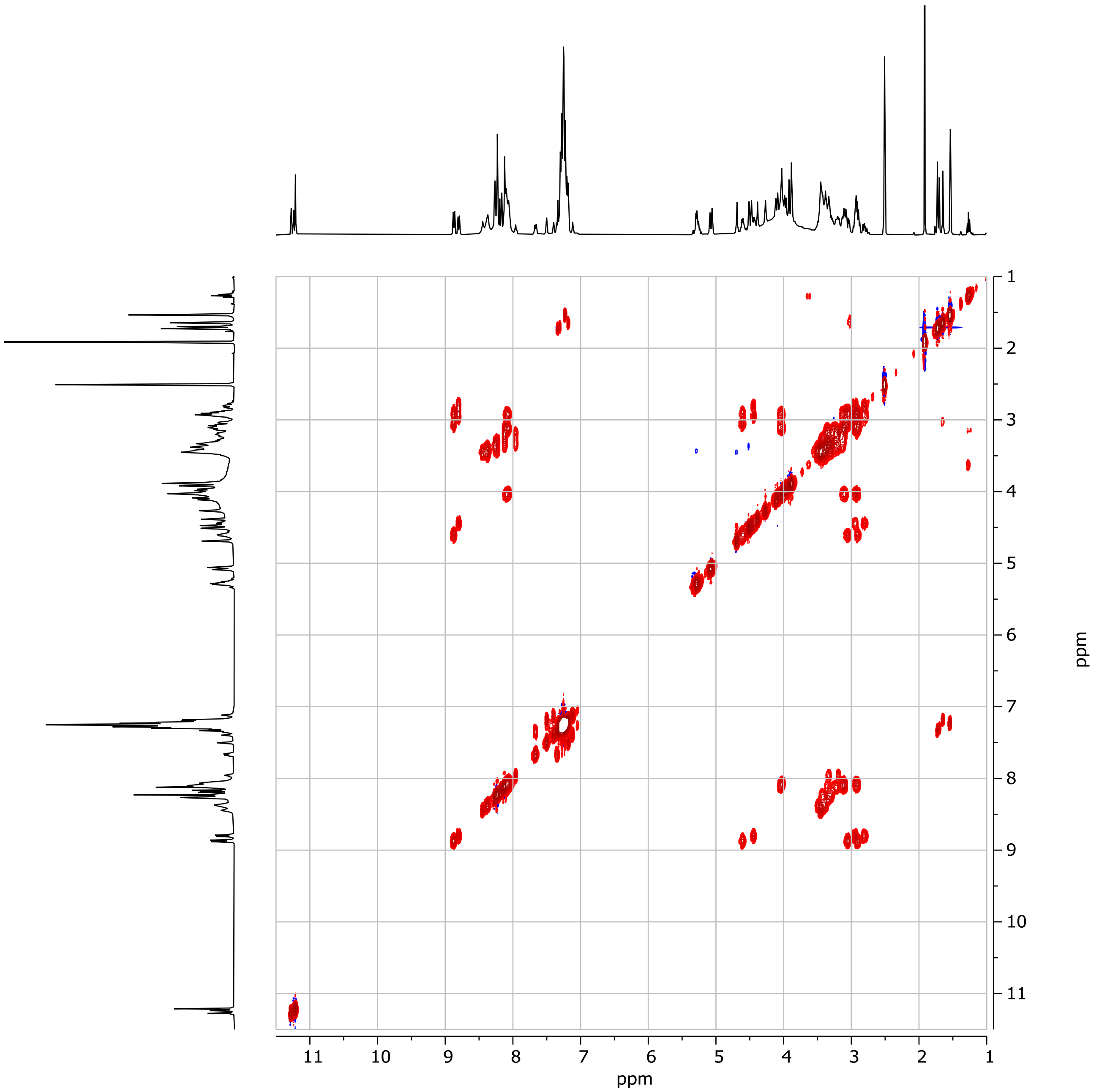

Figure S20: TOCSY NMR (400 Mz, DMSO, T = 300K) of Ffat.

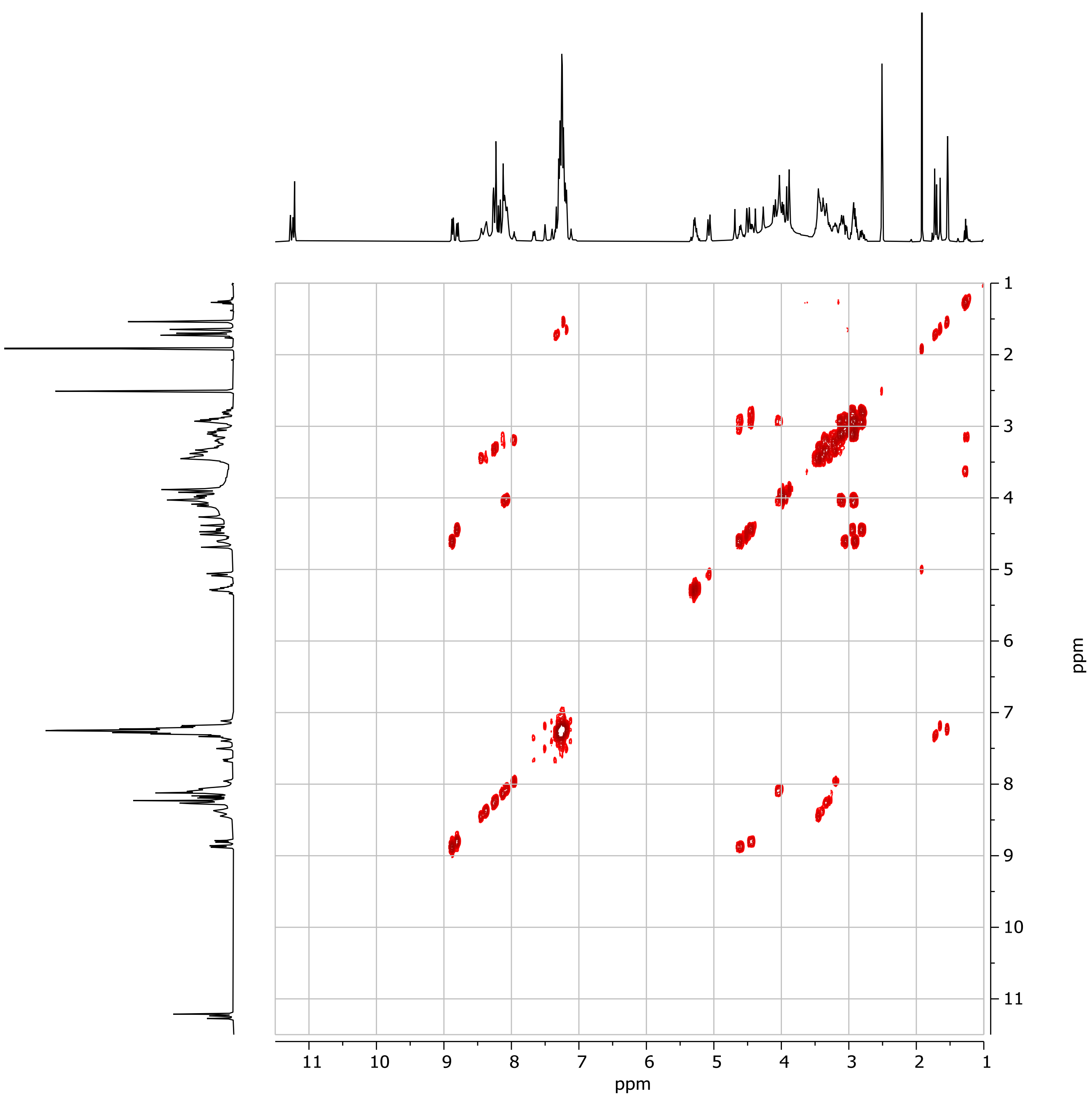

Figure S21: COSY NMR (400 Mz, DMSO, T = 300K) of Ffat.

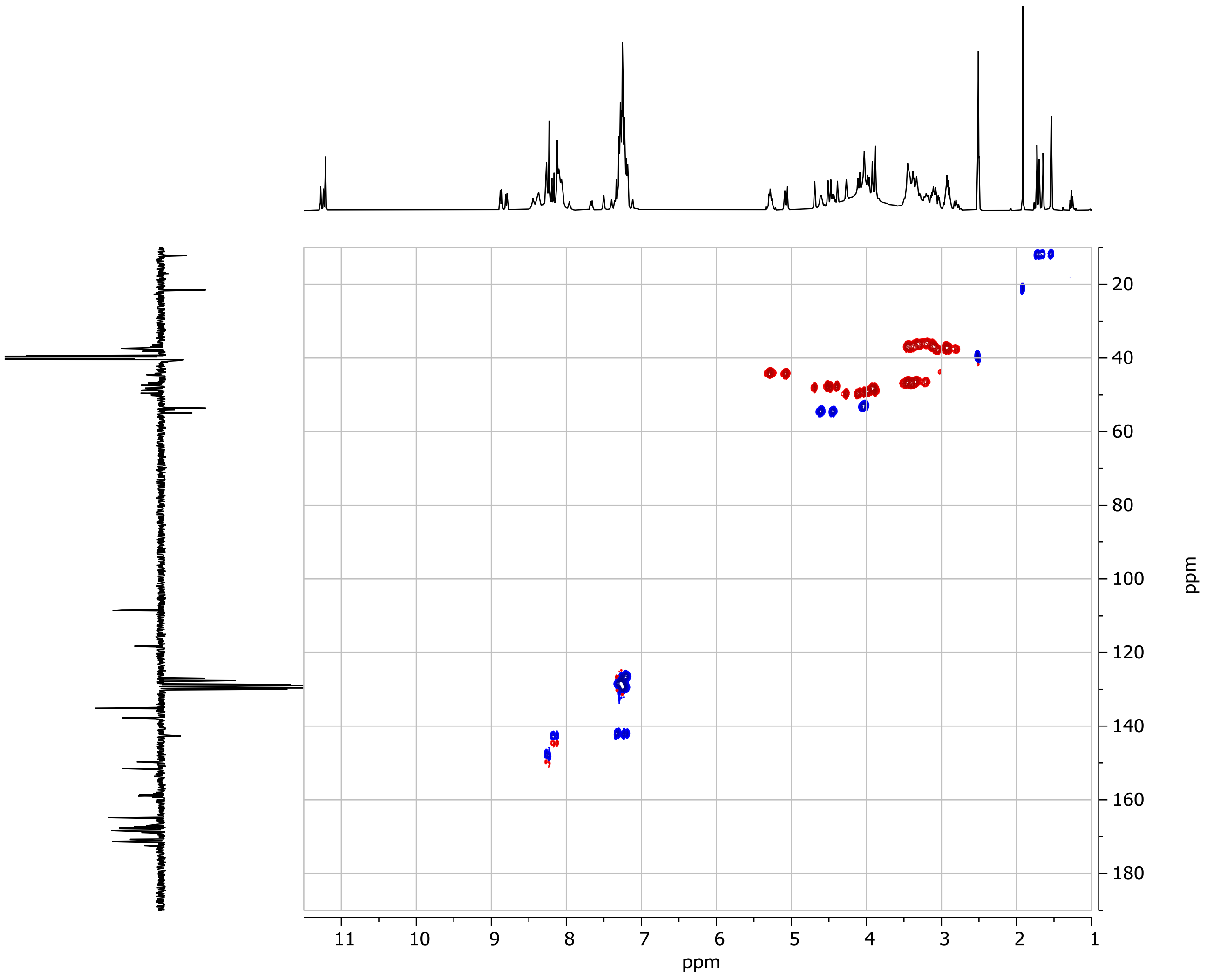

Figure S22: HSQC sp^2 NMR (400 Mz, DMSO, T = 300K) of Ffat.

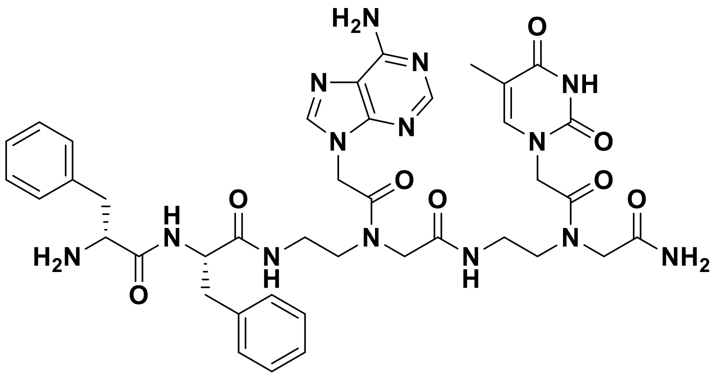

fFat

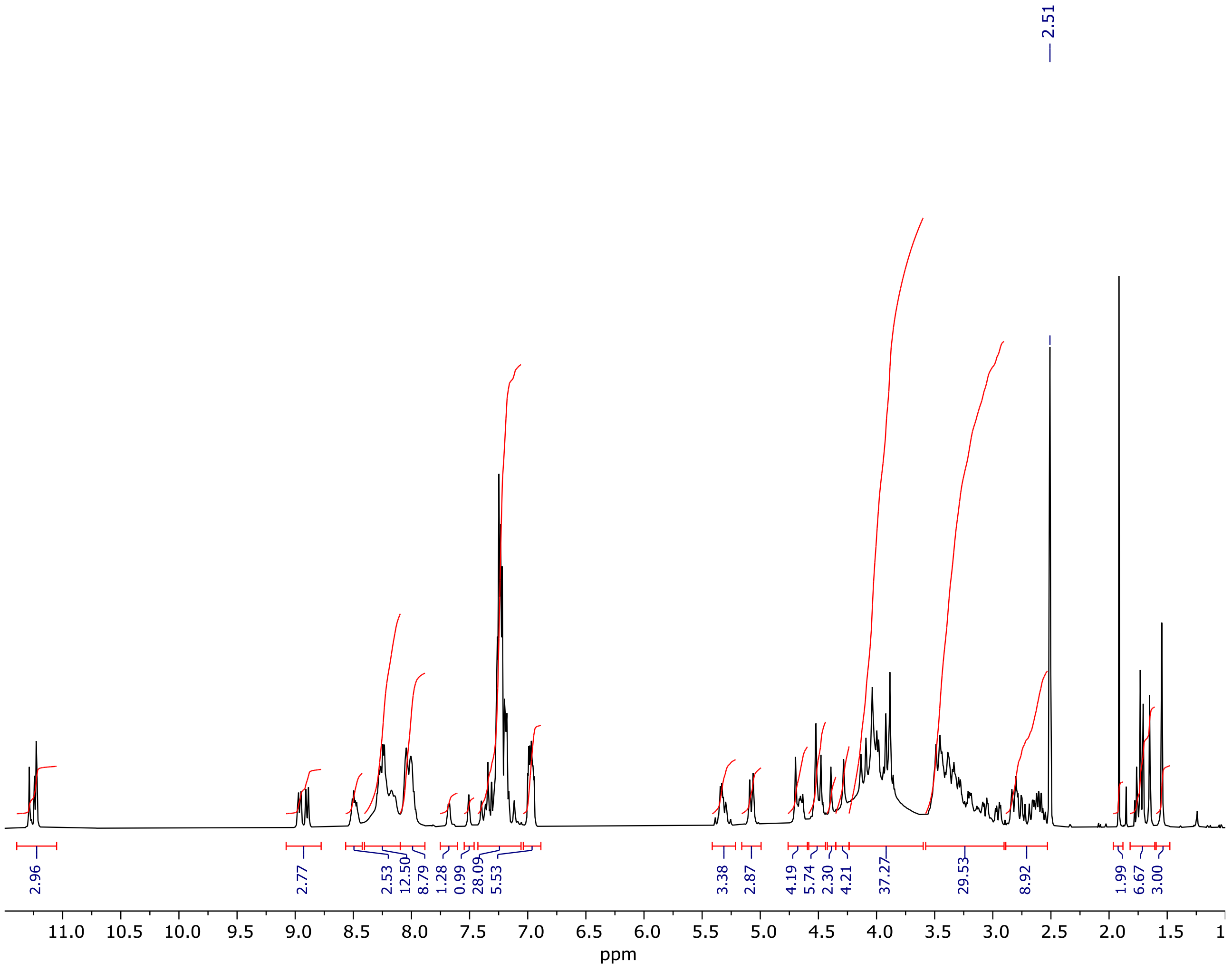

Figure S23: The chemical structure of the molecule and ^{1}H NMR (400 MHz, DMSO, T = 300K) of fFat.

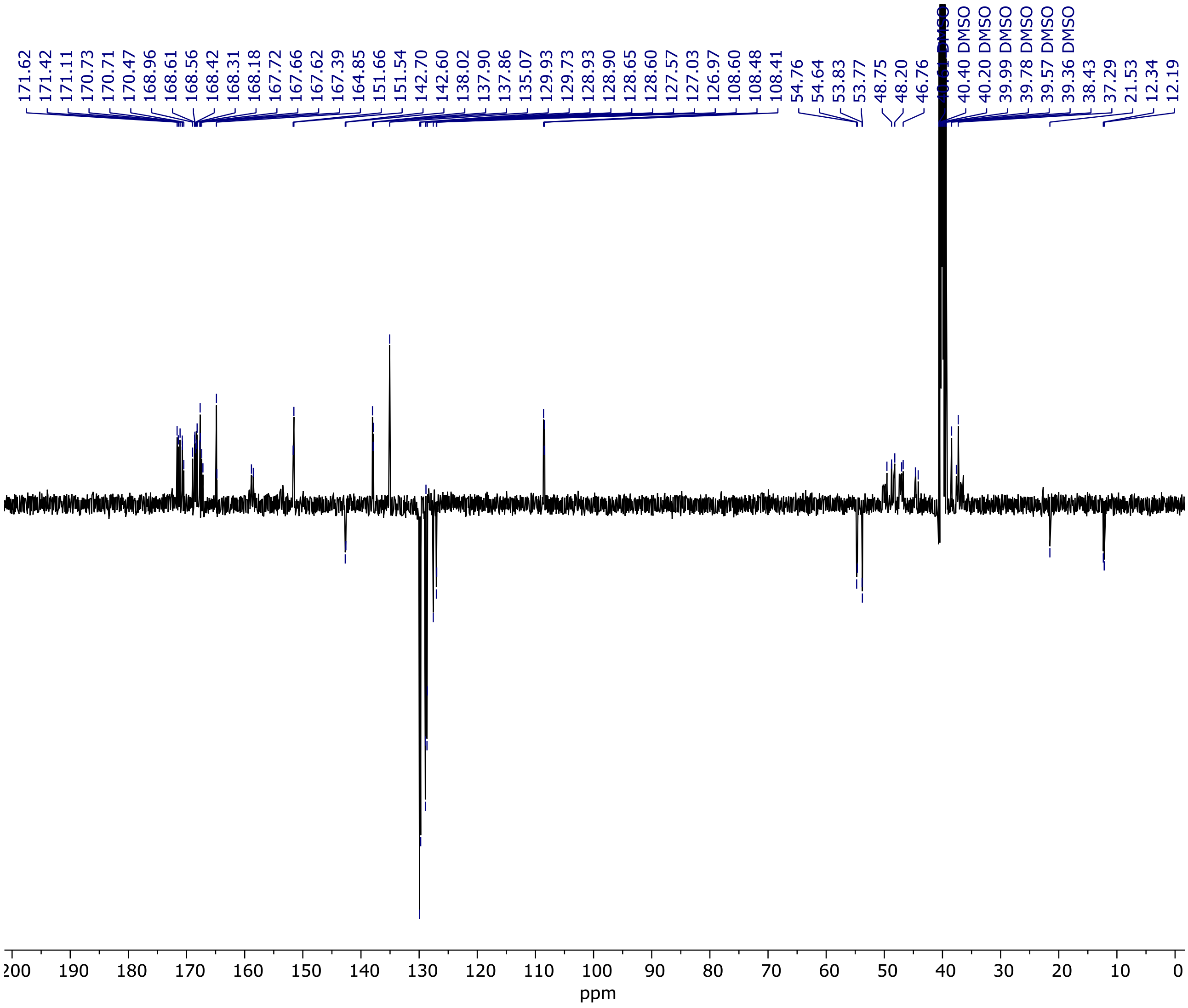

Figure S24: ^{13}C NMR-apt (100 MHz, DMSO, T = 300K) of fFat.

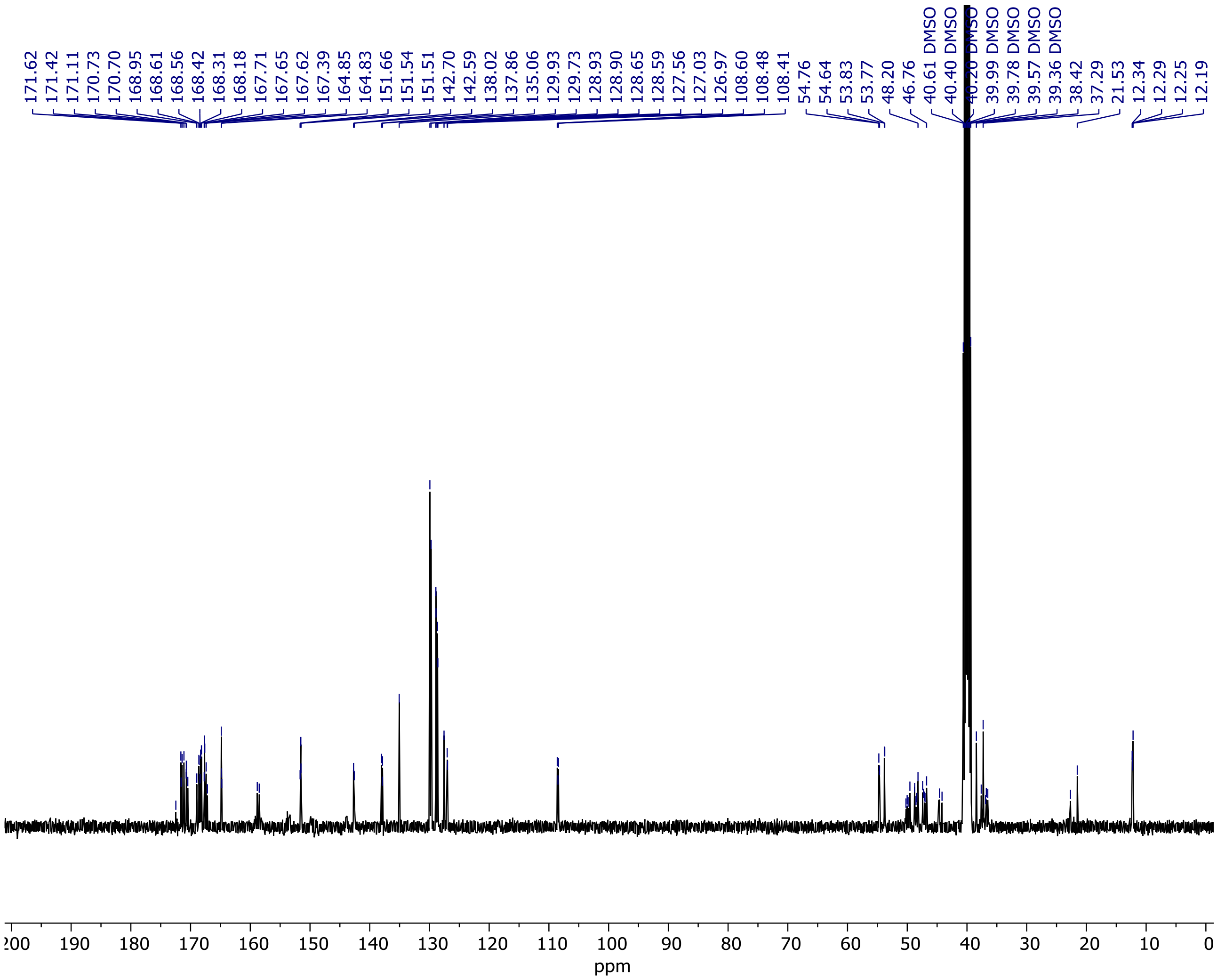

Figure S25: ^{13}C NMR (100 MHz, DMSO, T = 300K) of fFat.

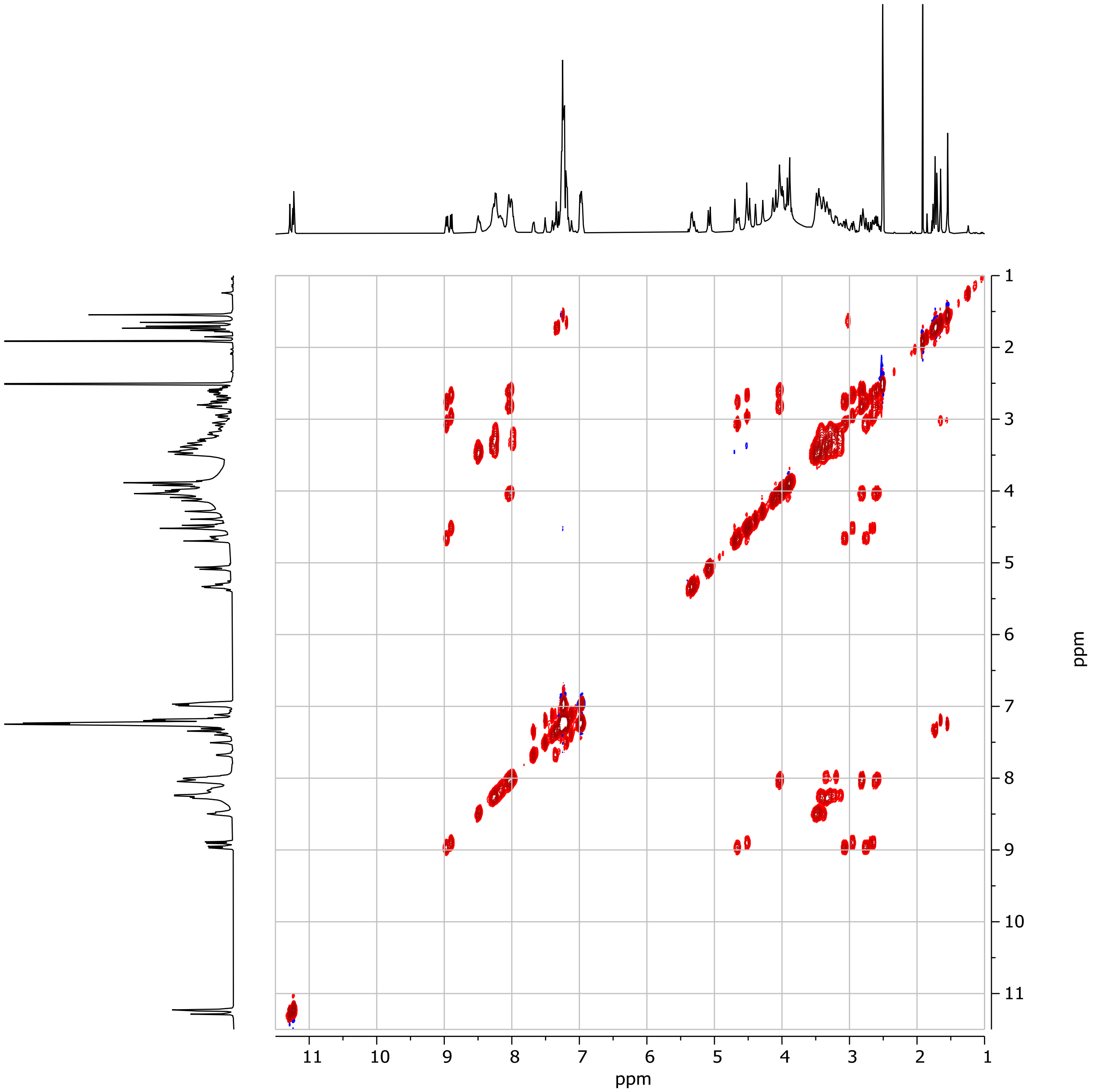

Figure S26: TOCSY NMR (400 Mz, DMSO, T = 300K) of fFat.

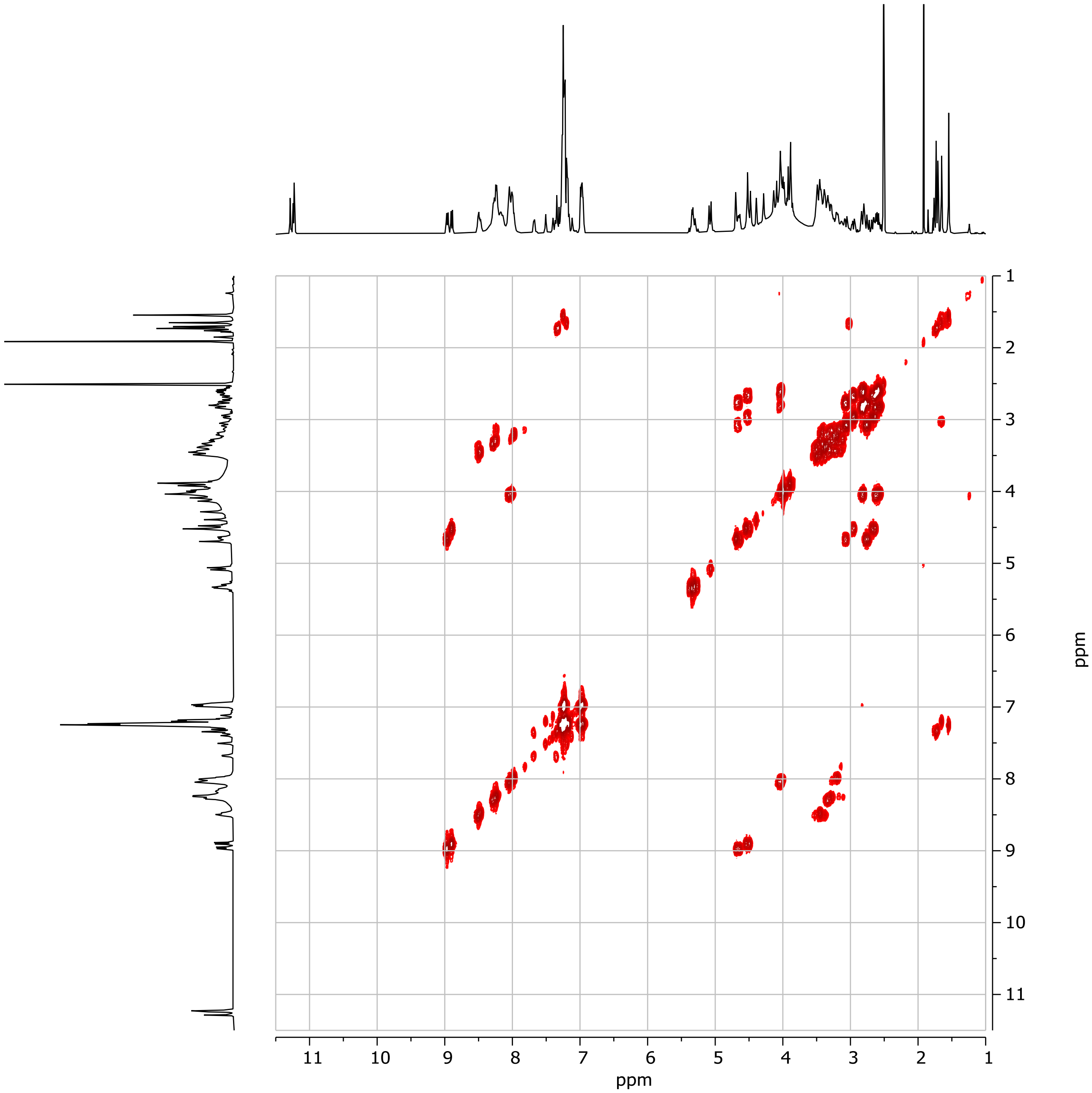

Figure S27: COSY NMR (400 Mz, DMSO, T = 300K) of fFat.

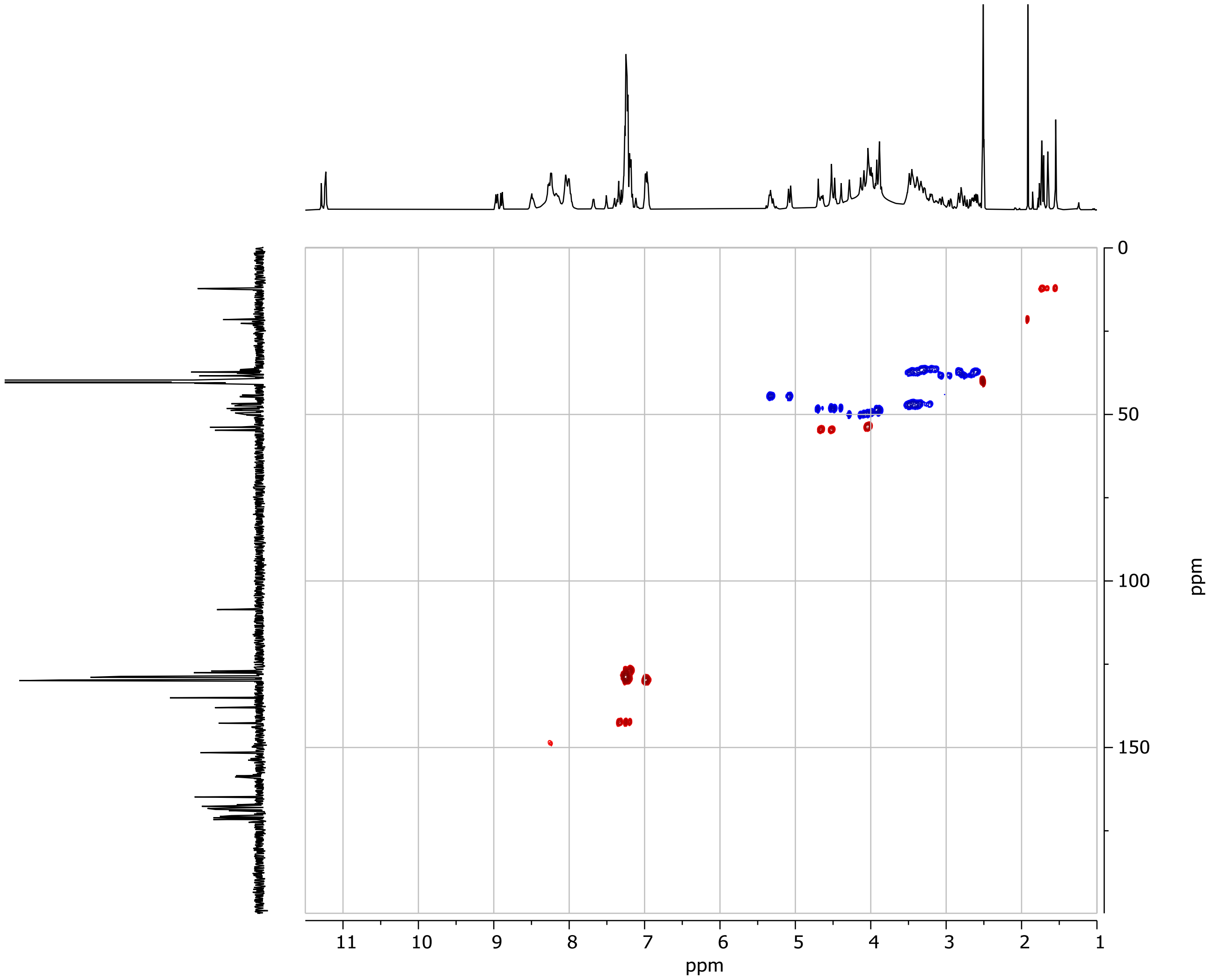

Figure S28: HSQC sp^2 NMR (400 Mz, DMSO, T = 300K) of fFat.

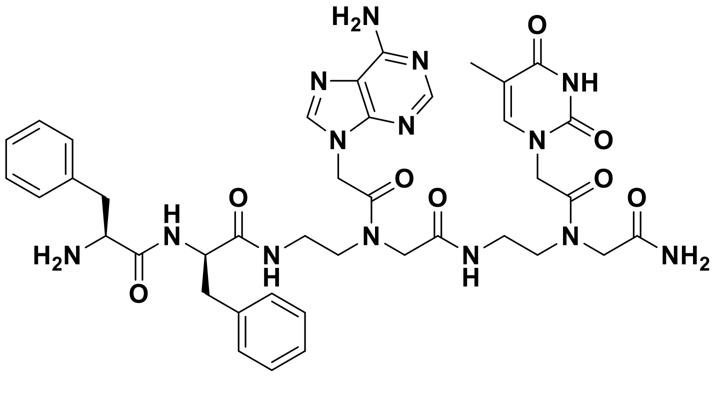

Ffat

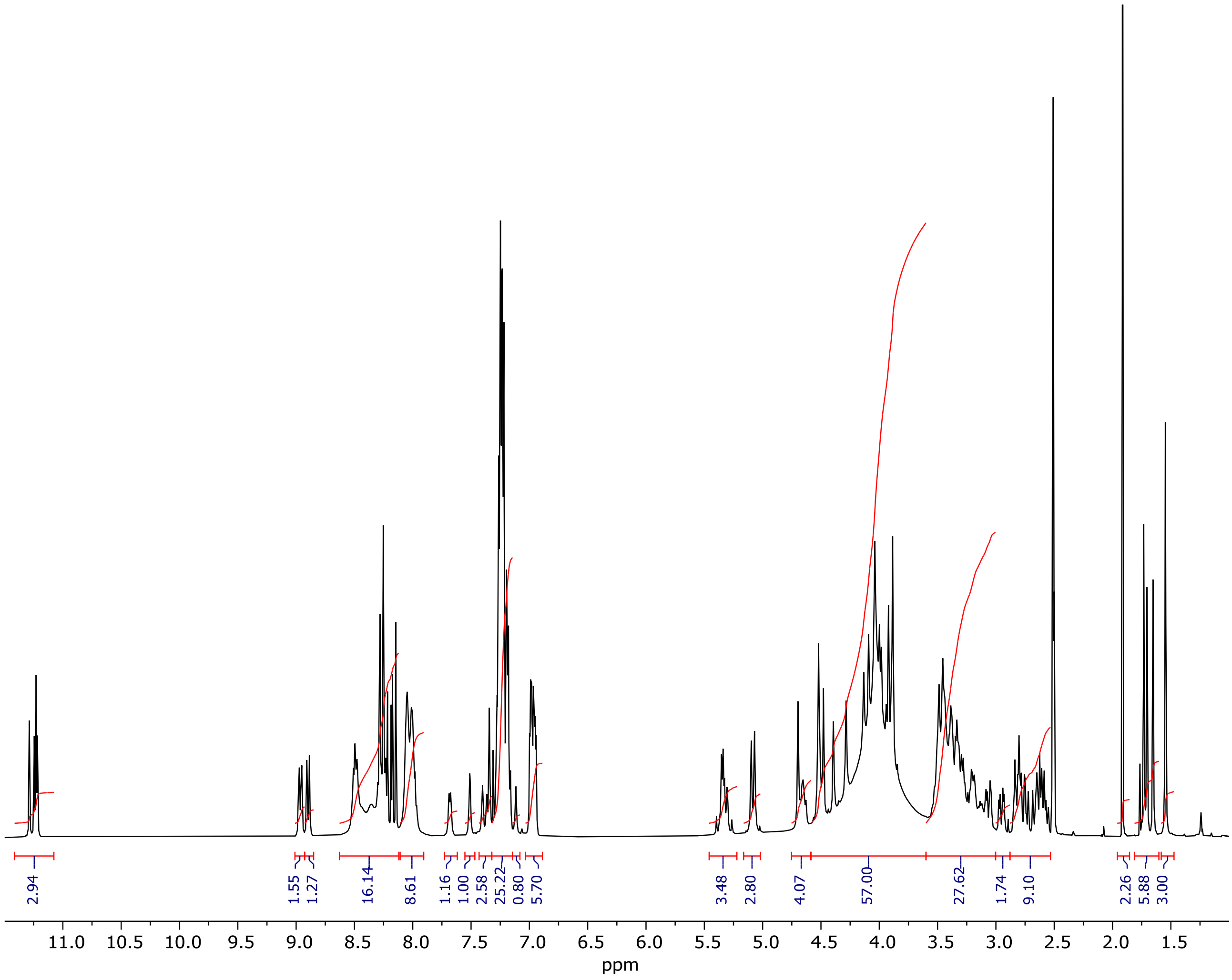

Figure S29: The chemical structure of the molecule and ^{1}H NMR (400 MHz, DMSO, T = 300K) of Ffat.

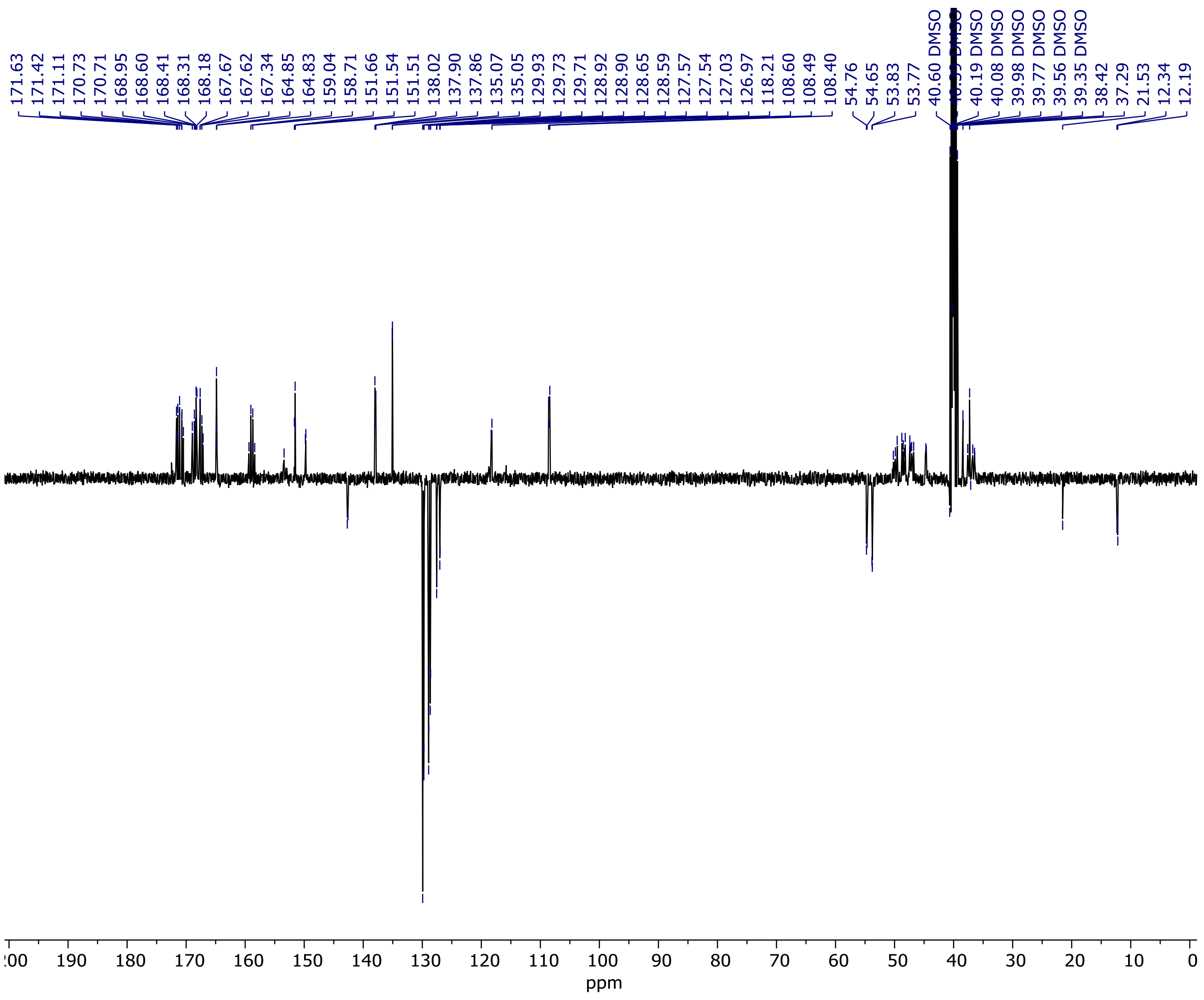

Figure S30: ^{13}C NMR-apt (100 MHz, DMSO, T = 300K) of Ffat.

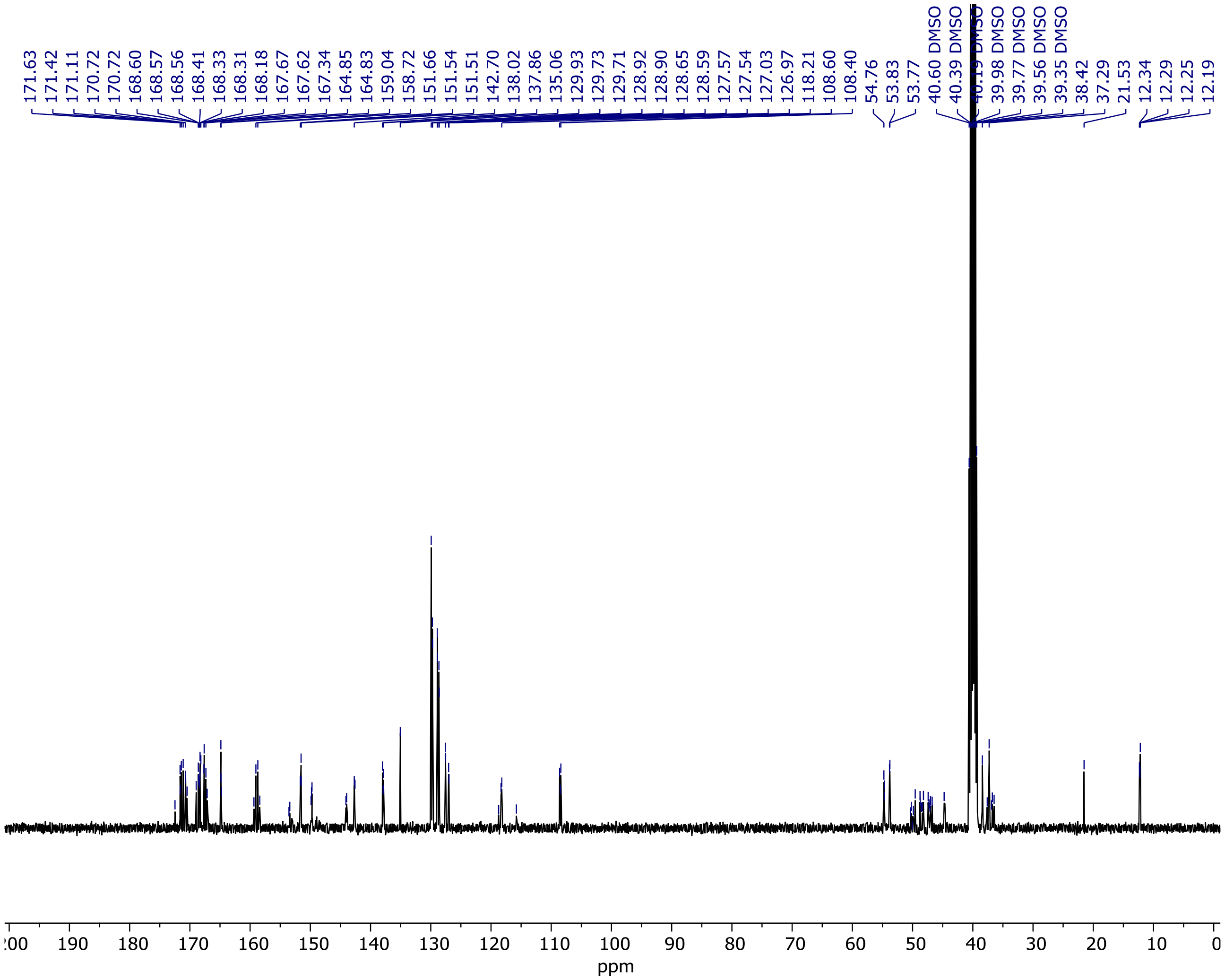

Figure S31: ^{13}C NMR (100 MHz, DMSO, T = 300K) of Ffat.

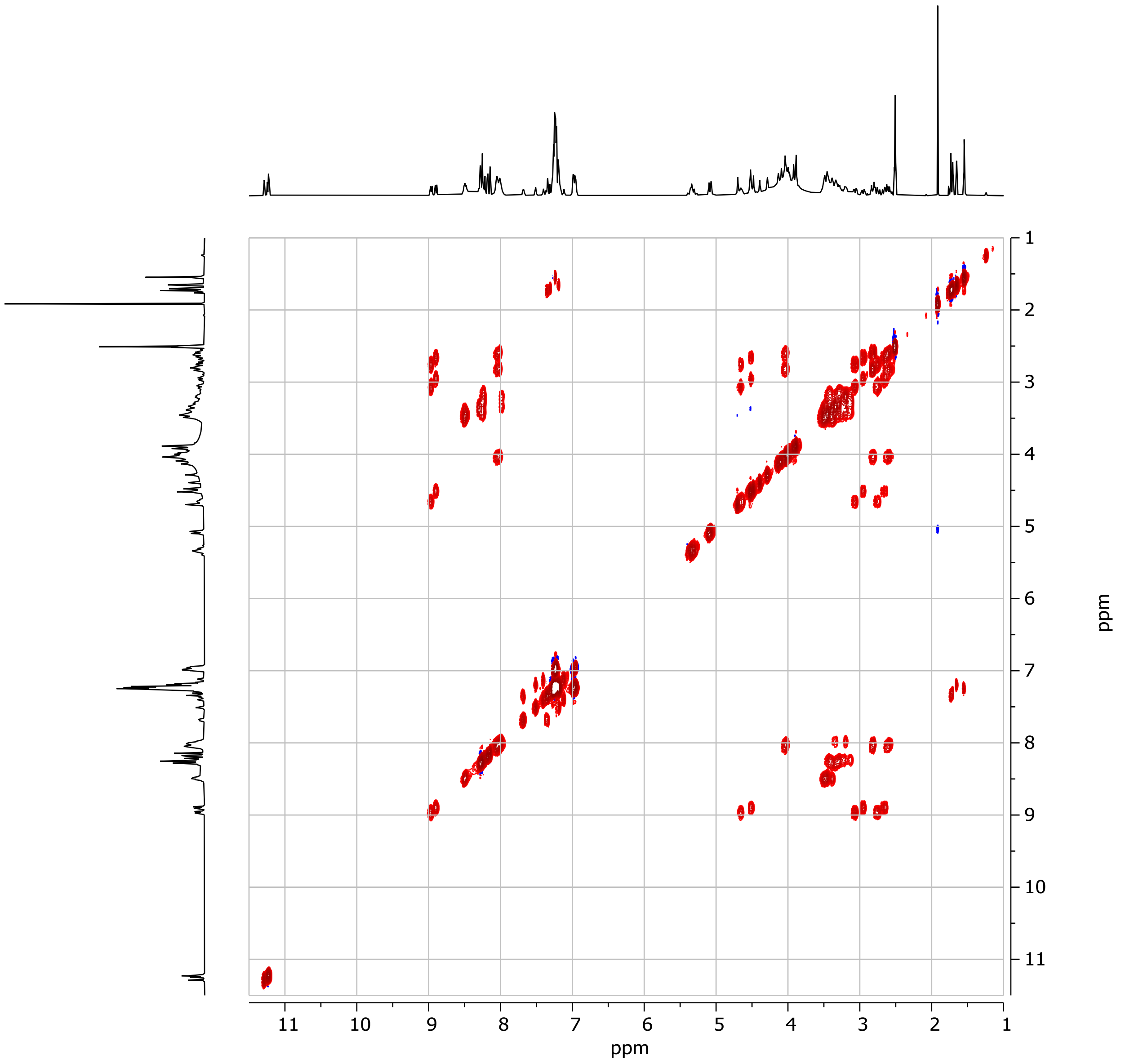

Figure S32: TOCSY NMR (400 Mz, DMSO, T = 300K) of Ffat.

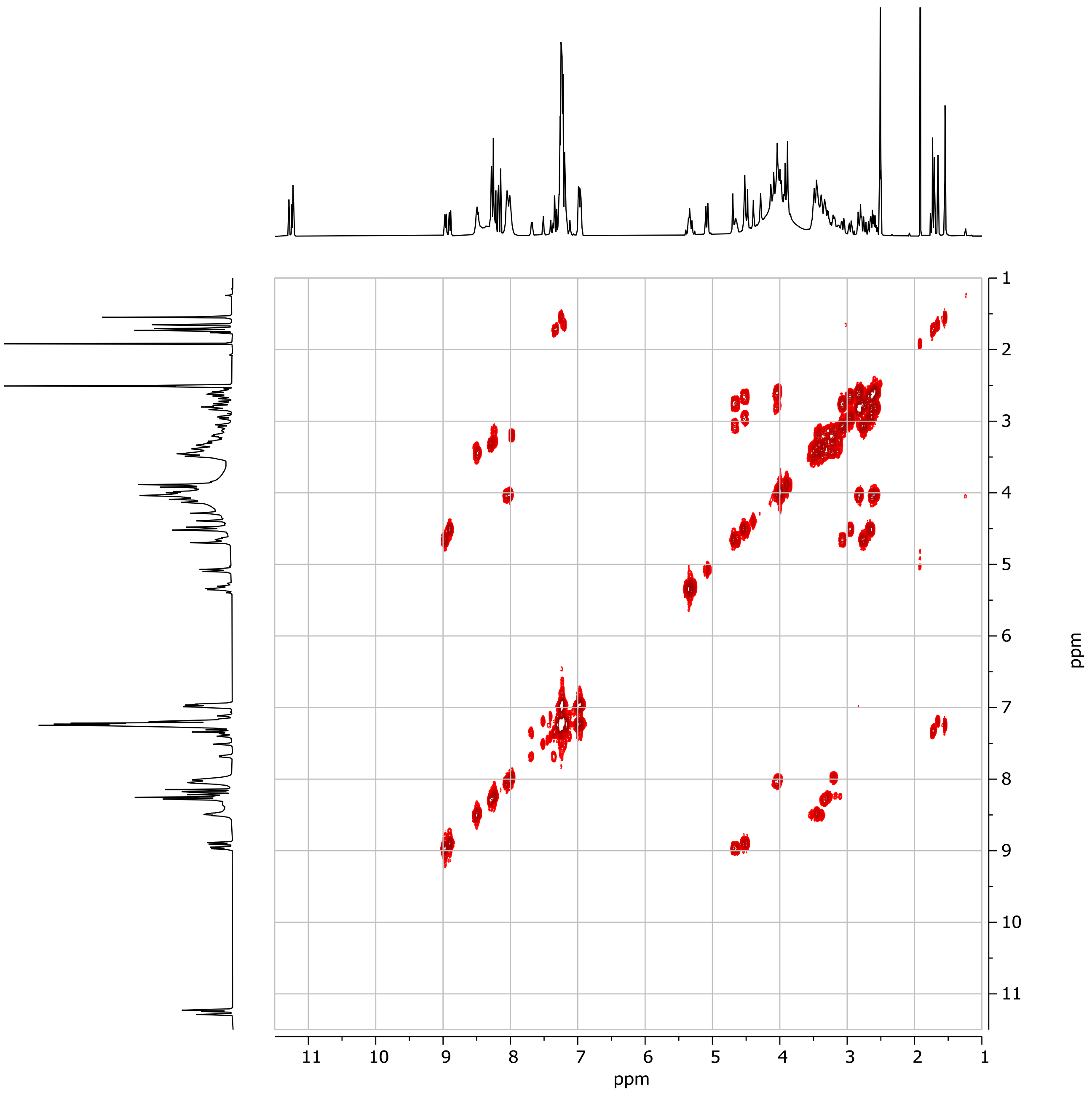

Figure S33: COSY NMR (400 Mz, DMSO, T = 300K) of Ffat.

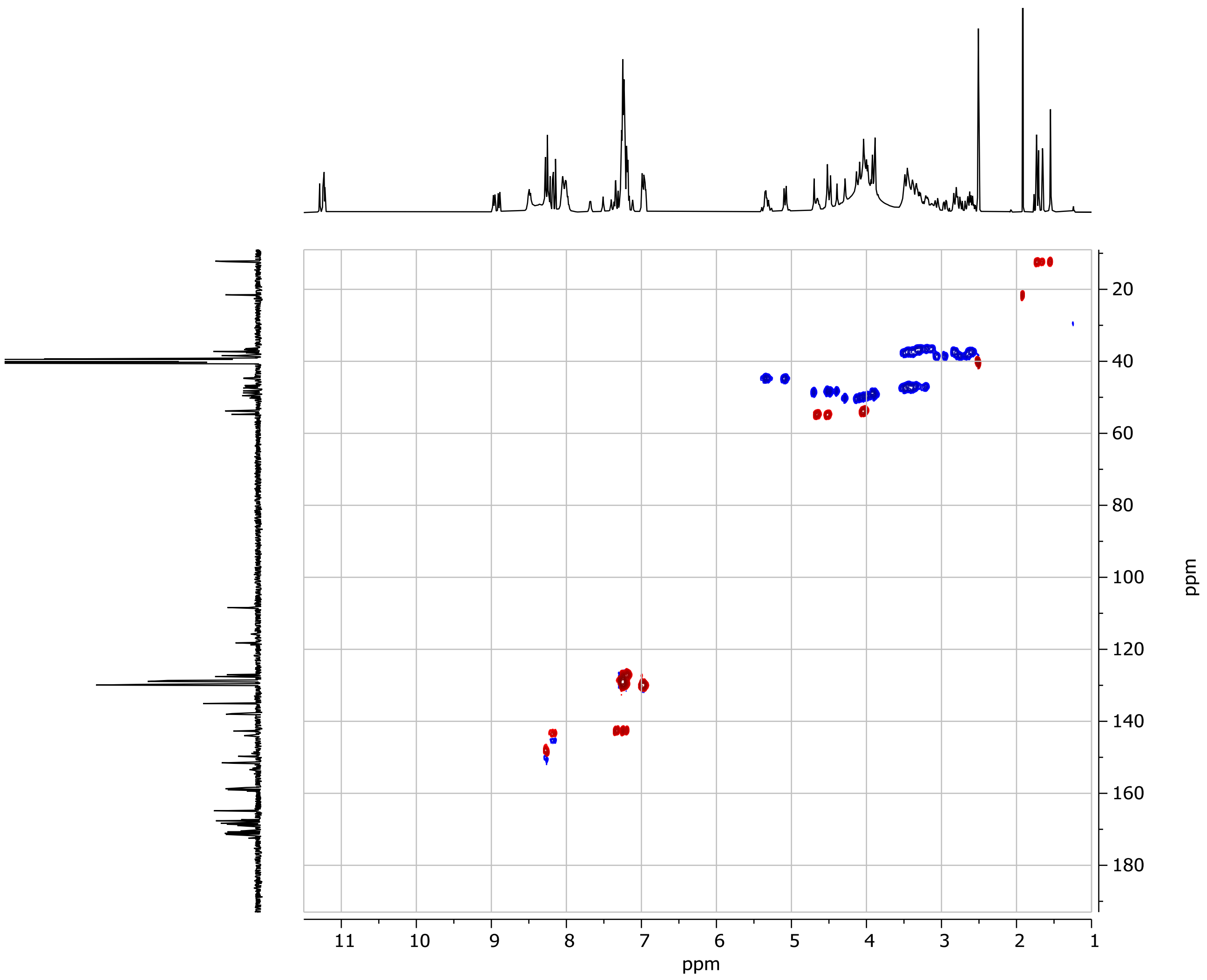

Figure S34: HSQC sp[2] NMR (400 Mz, DMSO, T = 300K) of Ffat.

1. Avitabile, C.; Diaferia, C.; Roviello, V.; Altamura, D.; Giannini, C.; Vitagliano, L.; Accardo, A.; Romanelli, A., Fluorescence and Morphology of Self-Assembled Nucleobases and Their Diphenylalanine Hybrid Aggregates. *Chem-Eur J* **2019,** *25* (65), 14850-14857.
2. Mosseri, A.; Sancho-Albero, M.; Leone, M.; Nava, D.; Secundo, F.; Maggioni, D.; De Cola, L.; Romanelli, A., Chiral Fibers Formation Upon Assembly of Tetraphenylalanine Peptide Conjugated to a PNA Dimer. *Chem-Eur J* **2022,** *28* (37).

3. Kralj, S.; Bellotto, O.; Parisi, E.; Garcia, A. M.; Iglesias, D.; Semeraro, S.; Deganutti, C.; D'Andrea, P.; Vargiu, A. V.; Geremia, S.; De Zorzi, R.; Marchesan, S., Heterochirality and Halogenation Control Phe-Phe Hierarchical Assembly. *Acs Nano* **2020,** *14* (12), 16951-16961.
